# Emergent Phototactic Responses of Cyanobacteria under Complex Light Regimes

**DOI:** 10.1128/mBio.02330-16

**Published:** 2017-03-07

**Authors:** Rosanna Man Wah Chau, Devaki Bhaya, Kerwyn Casey Huang

**Affiliations:** a Department of Bioengineering, Stanford University, Stanford, California, USA; b Department of Plant Biology, Carnegie Institution for Science, Stanford, California, USA; c Department of Microbiology and Immunology, Stanford University School of Medicine, Stanford, California, USA; UCSD; University of Hawaii-Manoa

## Abstract

Environmental cues can stimulate a variety of single-cell responses, as well as collective behaviors that emerge within a bacterial community. These responses require signal integration and transduction, which can occur on a variety of time scales and often involve feedback between processes, for example, between growth and motility. Here, we investigate the dynamics of responses of the phototactic, unicellular cyanobacterium *Synechocystis* sp. PCC6803 to complex light inputs that simulate the natural environments that cells typically encounter. We quantified single-cell motility characteristics in response to light of different wavelengths and intensities. We found that red and green light primarily affected motility bias rather than speed, while blue light inhibited motility altogether. When light signals were simultaneously presented from different directions, cells exhibited phototaxis along the vector sum of the light directions, indicating that cells can sense and combine multiple signals into an integrated motility response. Under a combination of antagonistic light signal regimes (phototaxis-promoting green light and phototaxis-inhibiting blue light), the ensuing bias was continuously tuned by competition between the wavelengths, and the community response was dependent on both bias and cell growth. The phototactic dynamics upon a rapid light shift revealed a wavelength dependence on the time scales of photoreceptor activation/deactivation. Thus, *Synechocystis* cells achieve exquisite integration of light inputs at the cellular scale through continuous tuning of motility, and the pattern of collective behavior depends on single-cell motility and population growth.

## IMPORTANCE

The photosynthetic cyanobacterium *Synechocystis* sp. exhibits phototaxis that is dependent on the incident light wavelength through the action of various photoreceptors. In natural environments, cells experience a set of highly dynamic and complex light inputs, yet how cells transduce multiple or dynamic inputs into motion is unknown. In this study, we measured the phototactic behaviors of single cells and communities as a function of light intensity or when illuminated by combinations of lights of different wavelengths or incidence directions. Responses to a spectrum of light regimes revealed that *Synechocystis* sp. integrates information about the light environment to tune its phototactic response, which is likely generated by competition among photoreceptors and the degree of wavelength-regulated growth to sensitively control the direction and degree of movement.

## INTRODUCTION

Bacteria are able to sense and respond to multiple external cues in their local environment. These responses allow cells to move into optimal or away from suboptimal environments by using a variety of appendages, such as flagella or pili. The chemotactic networks responsible for transducing inputs into complex motility responses are among the most extensively studied of all biological systems (1–3). Much of the knowledge about signaling in bacteria comes from cellular responses to individual inputs, although in the environment cells must deal with combinations of dynamic and antagonistic cues. In photosynthetic microbes, light powers photosynthesis, but high light levels and UV irradiation can also be damaging to cells, and so such microbes have evolved multiple mechanisms to reduce damage and move into optimal light environments. One such behavioral response is phototaxis, by which cells detect a light source and move toward it (positive phototaxis) or away from it (negative phototaxis). This process has been extensively studied in the model unicellular cyanobacterium *Synechocystis* sp. PCC6803 (here, *Synechocystis*) (1, 4–8). Photosynthetic cells experience varied light intensities and wavelengths throughout the day, which depend on the local environment of the bacterium (e.g., soil versus water, or over the diel cycle). These wavelengths of light are differentially absorbed and used by the photosynthetic apparatus, which in turn affects the energetics and growth of the cell. Cells appear to sense the direction of light rather than a gradient of intensity (6, 9), but we have limited understanding of how light is sensed, whether cells measure flux in a graded manner, how multiple signals are integrated, and how simple or complex light inputs are transduced to the bacterial motility machinery to achieve directional movement.

In our *Synechocystis* strain (the Carnegie substrain; see Materials and Methods), phototaxis occurs through biased movement toward or away from an illumination source placed at an oblique angle to the surface (see Movie S1 in the supplemental material) (10, 11). On a moist agarose surface, cells move toward a white light source, and over time communities of cells in finger-like projections emerge from an initially homogeneous distribution of cells (11, 12). The extent of formation of the finger-like projections at a fixed time point after inoculation is a function of the initial inoculation density (12). Furthermore, light input is also an energy source that allows for cell growth and division, which impact collective motility behaviors over long time periods. Cells exhibit both spatial and population heterogeneity in their phototactic responses, and individual cells can respond within minutes to changes in light conditions (10). Movement directionality is determined only by the current light direction, rather than based on a long-term memory of previous conditions (10). Our previous measurements indicated that motility bias likely results from the polarization of pilus activity, yielding variable levels of movement in different directions (10), similar to that seen with *Pseudomonas aeruginosa* chemotaxis (13–15). A recent study that used the Moscow substrain of *Synechocystis* sp. observed directed movement toward the light source rather than biased random walk behavior. This difference has also been observed at the population level, wherein an entire colony of the Moscow substrain moved toward white light; however, during phototaxis of the Carnegie substrain, a significant fraction of the cells remained in the space occupied by the original inoculation (16). The current study utilized only the Carnegie substrain.

**FIG 1:**
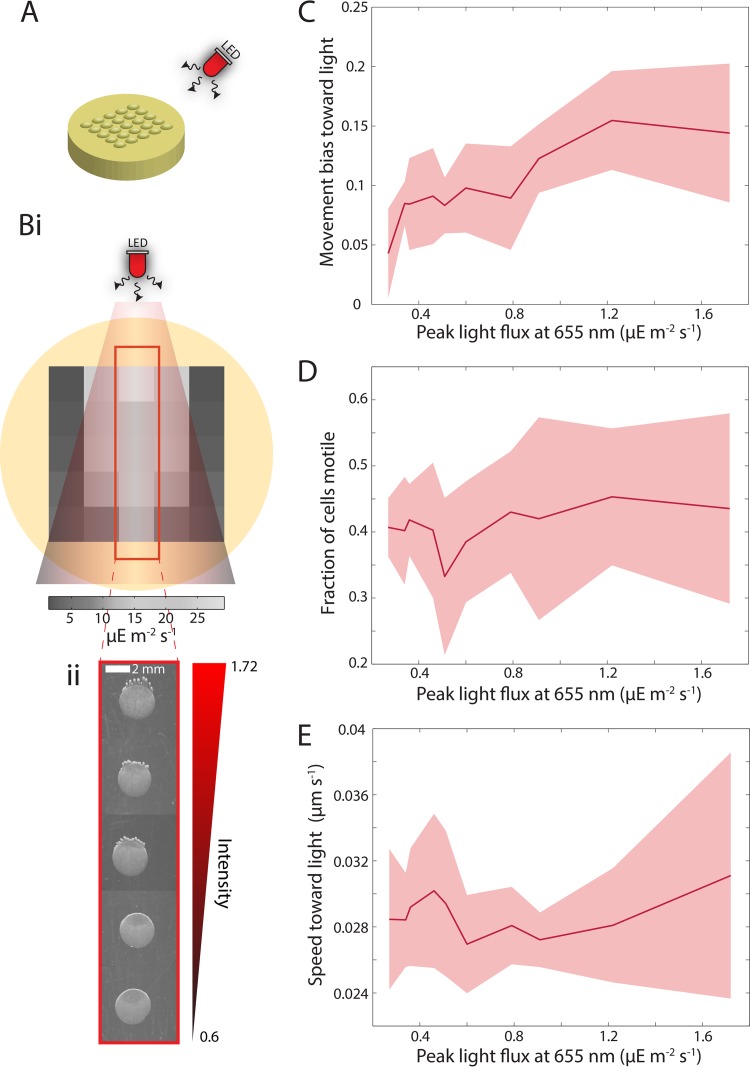
Intensity of red light illumination dictates phototactic bias. (A) *Synechocystis* cells were inoculated in a square grid pattern on a round petri dish and exposed to a red (660 nm) LED. The position of each spot on the plate determined the incident light intensity. (B, panel i) Color map of the integrated intensity of light from 400 to 700 nm at the center positions of each droplet of cells, with intensity decreasing at distances further from the LED. Intensity was measured in units of µE per square meter per second, with 1 µE = 10^−6^ mol photons. (ii) Snapshot of communities of cells along the central axis of the plate (red rectangle in panel i) imaged after 48 h in the presence of a red LED. Each circular community was inoculated onto one of the positions on the square grid in panel i. The gradient describes the peak intensity at 655 nm. Communities closer to the LED (top of the axis) exhibited longer finger-like projections than communities further from the LED (bottom of the axis). Similar behavior was observed in 7 independent experiments. (C to E) The movement bias (C) of individual cells increased with light intensity, while the fraction of cells that exhibit motility (D) (see Materials and Methods) and speed along the light axis (E) remained approximately constant. Single-cell measurements were obtained 6 h after inoculation. Error bars represent standard error of the mean (results from three independent experiments). The behavior of >500 cells was measured for each data point.

10.1128/mBio.02330-16.1 MOVIE S1  Time-lapse imaging of *Synechocystis* cells illuminated by a single red LED. LED illumination is from the top of the movie (as for Fig. 1 and 3). Cells appear to move in biased random walks toward the light. One microliter of cells from a culture with an OD_730_ of ~0.8 was placed in the center of a 50-mm plastic petri dish (BD Falcon) containing 0.4% (wt/vol) agarose in BG-11 medium. Upon adsorption of the inoculum onto the agarose, the plate was inverted and placed in front of a red LED (Roithner LaserTechnik GmbH; LED660N-03, 5 mm, 15 mW, 24° spread), such that the LED was positioned 50 mm from the center of the droplet (which had a diameter of 2.5 mm) at the level of the agarose. Imaging was conducted at the center of the droplet at 30°C, 24 h after being exposed to red light illumination. Download MOVIE S1, AVI file, 17.9 MB. Copyright © 2017 Chau et al. 2017 Chau et al. This content is distributed under the terms of the Creative Commons Attribution 4.0 International license.

In *Synechocystis*, incident light activates photoreceptors (5, 16–20), resulting in a signal transduction cascade that activates type IV pili (21–23), and motility is increased wherever cells have deposited extracellular polymeric substances (EPS) (11, 12), suggesting that EPS provides a medium for cell-cell communication to drive collective behaviors. Simulations based on a minimal biophysical model demonstrated that the combination of a biased random walk with motility-enhancing EPS secretion was sufficient to generate communities with similar fingering spatial patterns and on similar time and length scales as those determined from experimental data (12).

The phototactic response of cyanobacteria depends on the wavelength of the incident light. Light wavelengths ranging from red to green result in positive phototaxis, while cells do not move or exhibit negative phototaxis away from blue or UV light, which can damage DNA and other cellular components (1, 7, 8). Similar to plant phytochromes, some photoreceptors in *Synechocystis* exhibit two photoreversible conformational states upon absorption of light with a specific wavelength. TaxD1 (also known as PixJ1) is the major photoreceptor required for positive phototaxis. It is a cyanobacteriochrome that exhibits blue-green photoreversion and switches between a blue light-absorbing form (*P*_*b*_; *λ*_max_ = 425 to 435 nm) and a green light-absorbing form (*P*_*g*_; *λ*_max_ to 535 nm) (24). Some photoreceptor pairs also have overlapping absorption spectra (e.g., TaxD1 and PixD at 435 nm and TaxD1 and UirS at 535 nm), suggesting the potential for complementary or antagonistic roles in mediating positive and/or negative phototaxis (19, 24, 25). The molecular mechanism by which cells integrate a set of photoreceptor inputs is unknown.

In this study, we examined *Synechocystis* phototaxis in response to light of different wavelengths and intensities and to multiple inputs with different incidence directions or wavelengths. We determined that at the single-cell level, green light and red light promote positive phototaxis by increasing motility bias toward the light source in an intensity-dependent manner, while blue light inhibits motility altogether. *Synechocystis* cells integrate multiple light inputs of the same wavelength, resulting in motility along the vector sum of the light sources and altered step sizes under illumination by competing light sources. Recovery of phototactic bias at the single-cell level after transient substitution of blue light for green light was much slower than the loss of bias when green light was removed, but this slow recovery could be suppressed by maintaining the green light input. Taken together, our results reveal how interactions among the components responsible for promoting or inhibiting phototaxis are transduced into a complex range of cellular and community behaviors.

## RESULTS

### Phototaxis under narrow-spectrum red light illumination is flux dependent.

We performed a standard phototaxis assay (6, 10–12, 16), wherein spots of cells were illuminated by a red light-emitting diode (LED) placed almost level with the surface of the agarose (8). Red light illumination was selected to stimulate positive phototaxis, primarily because there is only one known *Synechocystis* photoreceptor that responds to red light (8). Small volumes (1 μl) of exponentially growing *Synechocystis* cells were spotted onto a low-concentration (0.4%) agarose plate in a grid pattern, such that each spot was at a different distance and/or orientation relative to a directional, narrow-spectrum red LED (Fig. 1A). We directly measured light flux at these grid positions (see Materials and Methods), verifying that cells in spots farther from the LED experienced less light flux than cells closer to the LED, as did spots placed away from the central axis of the LED (Fig. 1B, panel i).

We examined five spots along the central axis (Fig. 1B, panel i, red box) 48 h after inoculation and observed a flux-dependent community response: the extent of formation of finger-like projections (“fingering”) was progressively reduced at lower incident fluxes (Fig. 1B, panel ii). We investigated how this graded phototactic behavior arose by analyzing the motility of single cells. To achieve a finer resolution in flux, we imaged 10 spots placed at increasing distances away from the red LED 6 h after inoculation. The five highest intensities tested were the same as those in Fig. 1B, with fluxes ranging from 0.6 to 1.72 μE m^−2^ s^−1^ (measured at 655 nm). We observed a flux-dependent increase in the movement bias of cells (Fig. 1C). However, the fraction of motile cells (Fig. 1D) and their average speed along the axis toward the LED (Fig. 1E) were approximately constant, suggesting that cells respond to different fluxes in the light environment solely by changing the degree of bias toward the light source.

### Cells integrate information from multiple light sources with different incidence directions.

**FIG 2:**
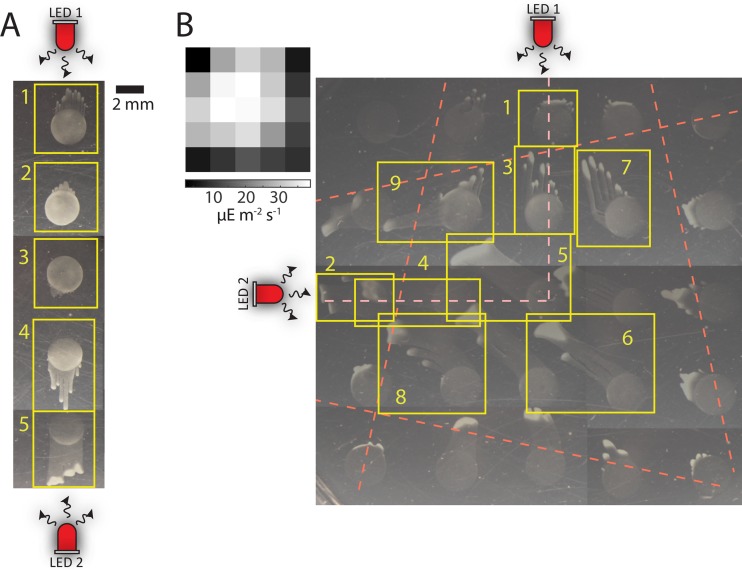
*Synechocystis* cells integrate information from multiple light sources with different incident directions. (A) A column of spots was exposed to two red LEDs on opposite sides of the dish at equal distances relative to the plate center. Communities were imaged after 48 h of light exposure. Spots in the center column closer to LED 1 (boxes 1 and 2) or 2 (boxes 4 and 5) moved toward the closer LED; the center community (box 3) exhibited only a slight degree of phototaxis toward LED 2. Similar behavior was observed in 7 experiments. (B) A square grid of spots on a round petri dish was exposed to two red LEDs oriented with 90° separation along the outside of the plate. Red lines represent the approximate extent of the cone of light illumination. Communities were imaged after 48 h of light exposure. Spots placed along the central axes exhibited characteristic fingering oriented toward the closer LED (boxes 1 to 4). Communities at the center of the dish or slightly farther from the LEDs moved along the vector sum of the two incident directions (boxes 5 and 6). Communities in boxes 7 and 8 first started moving toward the farther LED and then changed direction, presumably once they entered the cone of light from the closer LED. The community in box 9 bifurcated into fingers that moved toward each LED separately. Similar behavior was observed in 7 experiments. (Upper left inset) Color map of the estimated integrated intensity of light from 400 to 700 nm at the center positions of each droplet of cells. Intensities were estimated by combining the intensity matrix in Fig. 1B, panel i, with its transposed form, since the light meter could not simultaneously measure normally incident intensities from the two LEDs.

We hypothesized that cells are capable of sensing and integrating information from multiple light sources into a coordinated phototactic response. To test this hypothesis, we exposed *Synechocystis* cells to a combination of light sources with varying fluxes and from different directions. First, we illuminated cells with two red LEDs providing incident light from opposite directions (Fig. 2A). The LEDs were held along the periphery of a custom cylindrical chamber with a petri dish in the center of the chamber and were chosen to emit similar levels of light flux (as technically feasible). Phototactic behavior at five spots was observed, such that the center spot was equidistant from the two LEDs. After 48 h, spots 1 and 5 showed the highest degree of fingering, while spots 2 and 4 showed less fingering (Fig. 2A), which is consistent with results for experiments using one red LED (Fig. 1B, panel ii). Interestingly, these colonies displayed more extensive fingering than an equivalent colony exposed to only one LED (Fig. 1B, panel ii), suggesting that cells do not merely detect a difference between two incident fluxes. However, the center spot (spot 3) had no discernible fingering toward either LED (the very small finger at the bottom of the spot was likely caused by a slight difference in LED intensities). This finding suggests that signals from the two LEDs counteracted each other, resulting in a lack of phototaxis in either direction.

Next, we asked how cells respond to two perpendicular light sources (Fig. 2B; see also Fig. S1 in the supplemental material), which could provide conflicting directional signals. Several interesting and distinct behaviors were apparent. As expected, spots closest to the LEDs (boxes 1 and 2 in Fig. 2B) exhibited phototaxis toward the closer LED, although the fingering was not as extensive as with the spots slightly farther away along the incidence axes of the respective LEDs (boxes 3 and 4), suggesting that information from the second LED impacted phototaxis in the preferred direction. Most noticeably, inoculations located at the center (box 5) or in the lower right quadrant (e.g., box 6) exhibited fingering along the vector sum of the two LED light paths, indicating integration of the vector light signals. The behavior of the other spots that were in positions in which they were differentially exposed to the light sources as they exhibited fingering provided further insights into the dynamic behavior of phototaxing communities. Spots that were relatively close to one LED but initially outside the light cone of the closer LED changed finger direction over time, first moving toward the further LED, whose light cone was associated with higher flux, and then moving toward the closer LED as the flux from the closer LED increased (spots 7 and 8). Moreover, cells in spot 9 that were placed between the inner viewing half angles of the two LEDs underwent phototaxis toward both LEDs, with one subpopulation of cells moving toward LED 1 and another subpopulation moving toward LED 2 along the respective light axes (spot 9). Thus, communities respond to two light sources by integrating the incident fluxes in a dynamic manner.

10.1128/mBio.02330-16.2 FIG S1  Photograph of illumination from LEDs used for multiple-light-source experiments in a perpendicular arrangement. Download FIG S1, PDF file, 30.7 MB. Copyright © 2017 Chau et al. 2017 Chau et al. This content is distributed under the terms of the Creative Commons Attribution 4.0 International license.

### Light signals from different directions are integrated to either cancel light-based motility bias or induce superposition of the two biases.

To determine the origins of the dramatic range of macroscopic behaviors observed under a single light source or two light sources (either opposing or perpendicular), we performed single-cell time-lapse imaging and tracked motile cells within the central region of the central spot between two opposing or perpendicular LEDs, 24 h after the spots were placed on the agarose.

**FIG 3:**
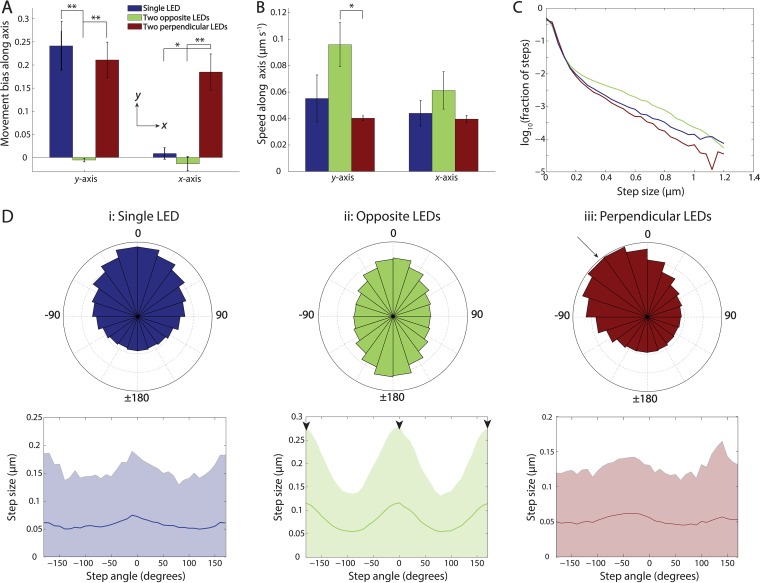
Light signals of equal intensity from opposite directions cancel light bias, while perpendicular signals induce a superposition of the two biases. (A) Movement bias along the *x* and *y* directions in the presence of various LED orientations. Motility bias was approximately 0 in both directions for two opposing LEDs; bias along the *x* and *y* axes in the presence of two perpendicular LEDs was similar to the *y*-direction bias of a single LED. Cells were imaged 24 h after inoculation. Error bars are standard error of the mean (*n* = 3 independent experiments). The statistical significance of differences in the means was determined using Student’s *t* test. *, *P* < 0.05; **, *P* < 0.01. (B) Single-cell speed along each axis did not significantly differ among all cases shown in panel A, except for the *y* direction for two opposing LEDs. *, *P* < 0.05. (C) Distribution of step sizes was similar for a single LED and two perpendicular LEDs, while cells took a higher fraction of larger step sizes in the presence of two opposing LEDs. The number of contributing cells was 951, 1,141, and 2,310 under each respective light condition. (D, top) By mapping steps to polar coordinates, distributions of single-cell step angles were found to be consistent with community behaviors. Concentric circles correspond to frequencies of 0.02, 0.04, 0.06, and 0.08. (Bottom) Step size as a function of step angle. Error bars indicate standard deviations of step sizes measured for steps in 10°-wide bins of angles. (i) In the presence of a single LED, cells take a larger fraction of steps toward the light, but step size is independent of step angle. (ii) In the presence of two opposing LEDs, cells increase the fraction of steps toward each LED, and step sizes are larger along the light axis (arrowheads). (iii) In the presence of two perpendicular LEDs, cells take a larger fraction of steps along the direction of the vector sum of the two light directions (arrow), and have a uniform distribution of step sizes as a function of step angles. The number of contributing cells was 951, 1,141, and 2,310 under each respective light condition.

In the community equidistant between two opposing LEDs that did not exhibit fingering (Fig. 2A), the movement bias along both the directions perpendicular (*x*) and parallel (*y*) to the light sources was close to zero (Fig. 3A, green bars), consistent with the fact that no fingering was observed when cells were exposed to opposing, equal intensity light. Nonetheless, cells maintained nonzero speeds in both the *x* and *y* directions (Fig. 3B), indicating that the opposing LEDs did not abolish motility. For the community exposed to two perpendicular LEDs, the bias was positive (toward the LEDs) in both directions, and the magnitude of bias was similar to that of a community at the same distance from a single LED along the incidence direction (Fig. 3A, red bars). Bias along the perpendicular direction was ~0 for a single LED (Fig. 3A, blue bars). Thus, motility bias at the single-cell level is consistent with the community-scale behavior.

As with a single LED employed at different fluxes (Fig. 1E), exposure to perpendicular LEDs did not affect the speed of motile cells relative to the speed with a single LED (Fig. 3B, red). However, exposure to opposing LEDs increased the speed of single cells, particularly in the directions of the light sources (Fig. 3B, green); this experiment is the only one that revealed a substantial increase in single-cell speed. This increase was consistent with the distribution of step sizes: cells took a higher fraction of longer steps in the presence of two opposing LEDs (Fig. 3C). The distribution of step angles was consistent with community behaviors and bias values (Fig. 3D, top), particularly in the case of two perpendicular LEDs, with cells most frequently stepping in the direction between the two LEDs (Fig. 3D, panel iii). The step-size distribution as a function of step angle was flat for a single or two perpendicular LEDs (Fig. 3D, panels i and iii). We were intrigued to find that, in the presence of two opposing LEDs, this distribution was nonuniform, with larger steps in the directions toward either LED (Fig. 3D, panel ii). These results for the two opposing LEDs are consistent with the faster motion of cells under this light condition (Fig. 3B) and suggest that the competing effects of the two opposing LEDs increase cell motility.

### Green light promotes phototaxis in a flux-dependent manner, while blue light stimulates growth but inhibits motility.

**FIG 4:**
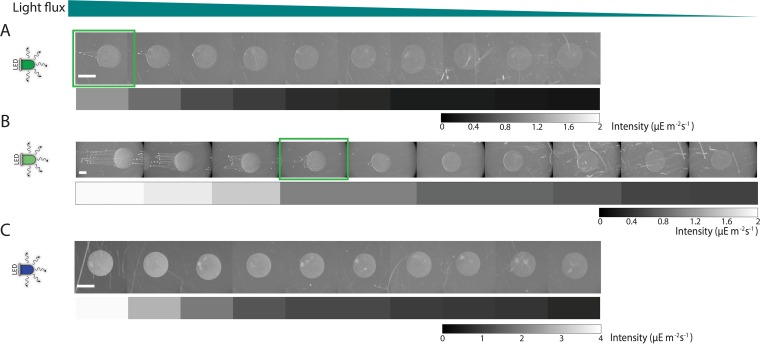
Green light promotes intensity-dependent phototaxis, while blue light stimulates *Synechocystis* growth but does not induce phototaxis. (A) Under illumination from a low-intensity green LED, communities experienced intensities ranging from 0.35 to 1.38 µE m^−2^ s^−1^ (measured at 535 nm). Communities were imaged after 48 h. Community movement toward the LED increased with increasing light intensity. The brightness of the communities indicated that there was also more cellular proliferation at higher intensities. (B) Under illumination from a high-intensity green LED, communities experienced intensities ranging from 0.51 to 1.97 µE m^−2^ s^−1^ (measured at 535 nm). Communities were imaged after 48 h. Movement toward the LED increased with increasing intensity, as shown in panel A, with the boxes highlighted in green experiencing similar incident intensities and exhibiting similar phototaxis. (C) Under blue light illumination, communities experienced intensities ranging from 0.65 to 3.91 µE m^−2^ s^−1^ (measured at 435 nm). Communities were imaged after 48 h. There was no net movement toward the LED. Cellular proliferation increased with increasing incident intensity. Bar, 2 mm (all panels). Similar behavior was observed in 7 experiments.

Typical phototaxis experiments have used white light or a narrow range of wavelengths to elicit motility responses, although previous results have shown that *Synechocystis* has photoreceptors that are responsive to different wavelengths of light (8) and that cells can exhibit both negative and positive phototaxis (4). Therefore, we argued that characterizing responses to specific wavelengths at varying intensities or to different combinations of light could provide additional insight into how these responses are integrated at a cellular level. We probed the phototactic responses to green light (535 nm), which promotes positive phototaxis, and blue light (435 nm), which inhibits phototaxis, at different incident fluxes. To encompass a range of green light fluxes, we used an intermediate-brightness and a high-brightness LED and measured the behavior under illumination intensities (measured at 535 nm) from 0.35 to 1.38 and 0.51 to 1.97 µE m^−2^ s^−1^, respectively (Fig. 4A and B). After 48 h, communities grown in a higher flux of green light demonstrated more fingering (left side of Fig. 4A and B), similar to behavior observed under red light illumination (Fig. 1B, panel ii). This behavior could be attributed to a flux-dependent increase in motility bias, while there was little change in the motile fraction and speed (Fig. S2). Communities experiencing higher fluxes also appeared denser (Fig. 4A and B; compare leftmost and rightmost spots, which were spotted at equal cell densities). Thus, there was likely more cell growth and division over this time period at higher light fluxes, which may have been partly responsible for the longer fingers.

10.1128/mBio.02330-16.3 FIG S2  Intensity of green light illumination dictates phototactic bias. *Synechocystis* cells were inoculated along the central axis of a square petri dish and exposed to a green (535 nm) LED, in the same setup as shown in Fig. 1B. The position of each inoculum on the plate determined the incident light intensity. The snapshot of communities of cells along the central axis of the plate imaged after 48 h is shown in Fig. 4B. The motility characteristics of single cells were investigated by imaging the region of each community closest to the LED at 6 h after inoculation. The movement bias of individual cells (A) increased with light intensity, while the fraction of motile cells (B) remained approximately constant. The speed along the light axis (C) decreased to a small extent with light intensity. Error bars are standard error of the mean from 6 independent experiments. At least 450 cells were used for each measurement. Download FIG S2, PDF file, 0.1 MB. Copyright © 2017 Chau et al. 2017 Chau et al. This content is distributed under the terms of the Creative Commons Attribution 4.0 International license.

Under the range of blue light fluxes tested (from 0.65 to 3.91 µE m^−2^ s^−1^, measured at 435 nm), we observed no phototaxis (Fig. 4C). As observed with green light illumination (Fig. 4A and B), there appeared to be more cell growth and division at higher intensities of blue light (Fig. 4C). From single-cell tracking, it was apparent that there were very few motile cells at any flux (Fig. S3), indicating that the lack of phototaxis is due to complete inhibition of motility by blue light; this behavior is in contrast to motility behavior in the dark, in which cells exhibit unbiased motion (10). Thus, green and blue light can stimulate a broad range of community behaviors encompassing different phototactic biases and proliferation levels.

10.1128/mBio.02330-16.4 FIG S3  Cells under blue light illumination exhibit no phototactic bias. *Synechocystis* cells were inoculated along the central axis of a square petri dish and exposed to a blue (435 nm) LED, similar to the experiment shown in Fig. 1B. The position of each inoculum on the plate determined the incident light intensity. The snapshot of communities of cells along the central axis of the plate imaged after 48 h is shown in Fig. 4C. The motility characteristics of single cells were investigated by imaging the region of each community closest to the LED, 6 h after inoculation. Under blue light illumination, cells demonstrated no movement bias of individual cells (A), regardless of light intensity, while the fraction of motile cells (B) and speed (C) remained approximately constant. The movement bias and speed of cells were measured along both the light axis and the axis perpendicular to the light source. Error bars are standard error of the mean from 3 independent experiments. At least 150 cells were used for each measurement. Download FIG S3, PDF file, 0.1 MB. Copyright © 2017 Chau et al. 2017 Chau et al. This content is distributed under the terms of the Creative Commons Attribution 4.0 International license.

### Combinations of two wavelengths tune phototactic behavior via integration with growth.

**FIG 5:**
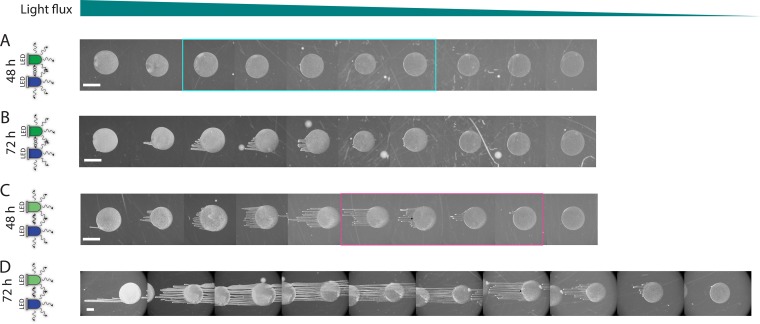
Multiple signals with different wavelengths prompt a complex range of behaviors that integrate promotion and inhibition of phototaxis with cellular proliferation. (A and B) *Synechocystis* communities were exposed to a combination of the low-intensity green LED used in Fig. 4A and the blue LED used in Fig. 4C. (A) After 48 h, phototaxis was almost completely inhibited, although the droplets highlighted in cyan exhibited a peripheral halo of cells in the direction of the LEDs. (B) After 72 h, a small degree of phototaxis was evident at intermediate intensities. (C and D) Communities were exposed to a combination of the high-intensity green LED used in Fig. 4B and the blue LED used in Fig. 4C. After 48 h (C), at all intensities, the brightness of the communities indicated that cells underwent more proliferation than in the presence of either green or blue light alone. At the higher intensities of blue and green light, phototaxis was inhibited relative to green LED illumination alone. At moderate intensities (highlighted in the pink box), phototaxis was intensity dependent and communities exhibited more movement toward the light than under green light alone. This pattern of phototaxis was still present after 72 h (D). Bar, 2 mm (all panels). Similar behavior was observed in 7 experiments.

Given that cells can integrate signals from two red LEDs to determine their phototactic behaviors (Fig. 2 and 3), we next queried how they respond to antagonistic or conflicting light signals. The combinations of two different wavelengths with the same incidence direction could result in behaviors that are not easily predicted from the responses under each wavelength separately. In particular, given the strong inhibition of motility under blue light, we conjectured that the inhibitory effects of blue light illumination would be dominant over green light promotion of phototaxis. To test this, we simultaneously exposed inoculations to a range of blue and green light fluxes, with a blue LED (0.65 to 3.91 µE m^−2^ s^−1^) in combination with either an intermediate- or high-intensity green LED (0.35 to 1.38 and 0.51 to 1.97 µE m^−2^ s^−1^, respectively) (as for the experiment shown in Fig. 4). After 48 h, the communities exhibited a combination of the behaviors observed under each wavelength alone. For cells that experienced the highest fluxes of blue light for both green LED (high- or low-intensity) configurations, the extent of phototaxis was reduced (Fig. 5) compared to when only green light was present (Fig. 4A and B). The degree of phototaxis initially increased with decreasing flux, and then it decreased at the farthest spots (Fig. 5). Interestingly, inoculations at intermediate/low fluxes of green and blue light formed finger-like projections that were not evident with corresponding inoculations exposed to a single green LED (Fig. 5C, pink box). Those communities also appeared denser, suggesting that the promotion of proliferation by blue light may result in increased motility, consistent with our previous observations of more rapid fingering and larger fingers at higher cell densities (12).

Comparison of the effects of the same panel of blue light fluxes combined with the lower (Fig. 5A and B) and higher (Fig. 5C and D) ranges of green light fluxes showed that the inhibition of phototaxis at higher blue flux was dependent on the incident green light flux; positive phototaxis could be recovered with higher green flux for the same incident blue flux (compare leftmost spots in Fig. 5A and B with Fig. 5C and D). In addition, the lag time before significant finger formation was shorter with the range of higher incident green light, as evident from images taken after 48 and 72 h (Fig. 5C and D). There are two possible causes of this acceleration: (i) as with blue light, green light could contribute to enhanced cell growth (thereby enhancing community-level phototaxis), and (ii) there may be cross talk between the signaling pathways that promote and inhibit phototaxis within cells. It is likely that both factors are at play, given the behaviors we observed under two LEDs (Fig. 2 and 3). We therefore conclude that the phototactic response of a cell to multiple inputs is graded, rather than such a response having a dominant promoting or inhibitory component.

### Rapid inhibition of single-cell motility from blue light illumination followed by slow recovery.

**FIG 6:**
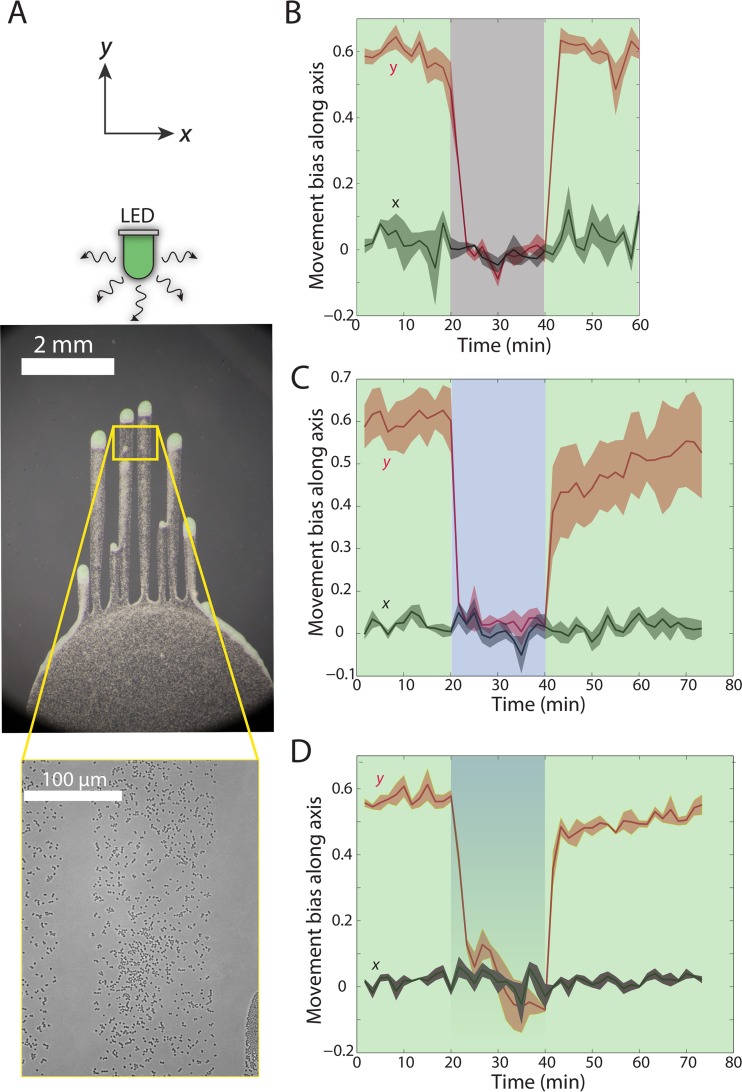
Blue light rapidly inhibits phototaxis, while recovery from inhibition occurs more slowly. Data for time zero denote results at the time of the first change in the light condition. (A) Representative phase-contrast images of the finger-like projections during times of imaging (shown in panels B to D). Cells were imaged in the mid-finger region (highlighted by the yellow box). (B) Cells were imaged for 20 min with incident green light (1.03 µE m^−2^ s^−1^ at 535 nm), after which time the light source was switched off for 20 min and then switched back on for 20 min. The motility bias parallel to the light quickly dropped to near zero once the light was turned off and then reestablished its original value within 100 s of light restoration. At least 150 cells were used for each measurement. Error bars represent the standard errors of the means for 4 independent droplets. (C) Cells were imaged for 20 min with incident green light, after which the green LED was switched off for 20 min and a blue LED (1.9 µE m^−2^ s^−1^ measured at 435 nm) was turned on, and then the two were switched back on and off, respectively, for 20 min. (i) Motility bias parallel to the light quickly dropped to near zero upon the first switch (green LED off). However, bias only recovered partially upon the second switch (green LED on) and then slowly approached the initial value over tens of minutes. At least 500 cells were used for each measurement with green light, and at least 150 cells were used for each measurement with blue light. Error bars show the standard error of the mean from 3 independent droplets. (D) As for panel C, except that the green LED was kept on throughout the experiment. (i) The decrease in motility bias upon blue LED illumination occurred more slowly than in panels B and C (over a few min). This bias recovered to a value much closer to the original value than the data shown in panel C, indicating that the inhibitory effect of blue light was ameliorated. At least 500 cells were used for each measurement with green light, and at least 150 cells were used for each measurement with blue and green light. Error bars indicate the standard error of the mean for 3 independent droplets.

We further investigated the competition between the activating and inhibitory pathways in *Synechocystis* cells by probing the effects of transient removal of the incident green light source or its replacement with blue light. Switching from green to blue or darkness should inactivate or deactivate, respectively, TaxD1 by converting the photoreceptor from the active *P*_*b*_ to inactive *P*_*g*_ state or by dark reversion to the inactive photoreceptor state (24). At the same time, the presence of blue light could activate photoreceptors that inhibit positive phototaxis (e.g., UirS, Cph2, CCry1-DASH [7, 16, 19, 26]). To enable comparisons with results of our previous studies (10), we grew communities that had finger-like projections and then measured the single-cell behavior in the midfinger region at least 24 h after inoculation of cells on the agarose (Fig. 6A).

First, we switched the green light source off for 20 min and then restored the light. As we previously observed with a broad-spectrum warm-white LED (10), cells lost their phototactic bias upon removal of the green light source but rapidly regained their original movement bias with the renewal of light within the minimal 100-s interval necessary to accurately quantify bias (Fig. 6B). Single-cell speeds along both axes dropped by ~40% in the dark (Fig. S4A), and the preswitch speeds were reestablished once the green LED was turned back on (Fig. S2). This observation was in contrast to cellular behavior in the dark after white light illumination, wherein speed was maintained in the dark (10). When we switched a red LED off for 20 min, bias quickly dropped to 0 (Fig. S5A), and the speed in the dark decreased to a lesser extent (Fig. S5B) than when green light was the original source (Fig. S4A).

10.1128/mBio.02330-16.5 FIG S4  The speed of cells is dependent on the instantaneous incident light wavelength. (A) Cells were imaged for 20 min with incident green light (Green 1), after which the light source was switched off for 20 min (Off) and then switched back on for 20 min (Green 2). The mean speeds of cells along both axes dropped by ~40% (*y*) and ~30% (*x*) in the absence of light, and preswitch speeds were reestablished once the green LED was turned back on. At least 150 cells were used for each measurement, and the standard errors of the mean are shown for 4 independent inoculations. ***, *P* < 0.001. (B) Cells were imaged for 20 min with incident green light (Green 1), after which the green LED was switched off for 20 min and a blue LED was turned on (Blue), and then the two were switched back on and off, respectively, for 20 min (Green 2). The speeds of cells along both axes dropped by ~40% in the presence of blue light; preswitch speeds were approximately reestablished once the green LED was turned back on. At least 500 cells were used for each measurement with green light, and at least 150 cells were used for each measurement with blue light. Error bars show the standard error of the mean from 3 independent inoculations. *, *P* < 0.05; **, *P* < 0.01. (C) Results of an experiment similar to that shown in panel B, except for the green LED being kept on throughout the experiment. The speeds of cells along both axes dropped by ~40% in the presence of blue light. Preswitch speeds were reestablished once the green LED was turned back on. Distributions were compiled using 3 independent inoculations. At least 500 cells were used for each measurement with green light, and at least 150 cells were used for each measurement with blue light. Error bars indicate the standard error of the mean from 3 independent inoculations. **, *P* < 0.01. Download FIG S4, PDF file, 0.3 MB. Copyright © 2017 Chau et al. 2017 Chau et al. This content is distributed under the terms of the Creative Commons Attribution 4.0 International license.

10.1128/mBio.02330-16.6 FIG S5  The change in behavior of cells upon light loss is dependent on the prior-experienced incident light wavelength. Using a similar strategy as in Fig. 6B and S2A, cells were investigated for changes in single-cell motility behavior upon removal and restoration of red (660 nm) light. Cells were imaged for 20 min with incident red light (Red 1, 0.91 µE m^−2^ s^−1^ at 655 nm), after which the light source was switched off for 20 min (Off) and then switched back on for 20 min (Red 2). (A) The movement bias of cells along the *y* axis decreased to approximately 0 within 100 s of red light removal, and bias was rapidly reestablished upon restoration of the red light. The movement bias of cells along the *x* axis remained approximately 0 throughout the experiment. (B) The mean speeds of cells along both axes dropped by ~20% (*y*) and ~10% (*x*) in the absence of light, and preswitch speeds were reestablished once the red LED was turned back on. At least 1,200 cells were used for each measurement, and error bars represent the standard error of the mean from 3 independent inoculations. *, *P* < 0.05; ***, *P* < 0.001. Download FIG S5, PDF file, 0.1 MB. Copyright © 2017 Chau et al. 2017 Chau et al. This content is distributed under the terms of the Creative Commons Attribution 4.0 International license.

We next switched from green light to an intermediate blue light flux for 20 min, which resulted in the rapid loss of phototactic bias (Fig. 6C) and a partial decrease in speed (Fig. S4B), as observed in the dark switch (Fig. 6B; Fig. S4A). However, full restoration of phototaxis upon renewal of green light required a much longer time; cells initially increased their motility bias over the first few minutes but did not regain their original motility bias even after 40 min (Fig. 6C). Switching from blue light back to green should activate TaxD1 and PixD by converting the photoreceptors to the active form that promotes phototaxis while allowing the inhibitory photoreceptors to revert back to their inactive, “dark” state.

Finally, we maintained green light illumination throughout the experiment and added intermediate blue light for 20 min. Loss of phototaxis occurred on a slower time scale (~5 min) (Fig. 6D), consistent with our other data indicating that inhibitory effects can be overcome by the presence of phototaxis-promoting signals (Fig. 5). The addition of blue light still reduced the speed (Fig. S4C). The time scale for recovery of motility bias was intermediate between the dark (Fig. 6B) and blue-light-only (Fig. 6C) conditions, with an initial, fast recovery to a level somewhat below the initial bias and a second, slow recovery phase (Fig. 6D). These results suggest that blue light-activated photoreceptors inhibit motility for some time and that dark reversion or loss of phototaxis inhibition takes place on a longer time scale than that of activation of phototaxis, consistent with previous measurements of the dark-reversion half-life of TaxD1 (>1 h) (24).

## DISCUSSION

Our study shows that phototactic bias in *Synechocystis* is intensity dependent under red and green light illumination, but the fraction of cells that are motile is independent of intensity, as is their speed in each direction, indicating that cells are primed for a change in light input that would require them to alter their motility bias. How do cells control this bias in an intensity-dependent manner? The maintenance of speed argues against a global increase in the pilus activity responsible for cellular locomotion (Fig. 1D; see also Fig. S2C in the supplemental material). Instead, our data suggest that pilus localization or activity control conserves the amount of pulling along the incidence direction; it is likely that the ratio of pulling toward versus away from the light dictates bias. A roughly equivalent fraction of pulling events must also occur perpendicular to the light, which may allow *Synechocystis* cells to tune their bias in response to changing conditions. Our data thus also predict that the fraction of photoreceptors that are active at any time is intensity dependent. Such behavior has been observed in the blue light flux-dependent activation of the PixD photoreceptor, through dissociation of the inactive PixD-PixE complex into its active components (27). The rapid changes in direction that we observed could be due directly to photoreceptor switching between active and inactive states, or to downstream components of the motility transduction pathway, such as competition between pili.

We previously observed a similar maintenance of speed under dark conditions after switching from white light illumination (10), indicating that light is at least transiently not required for cellular motility. However, our observations in the current study revealed that speed determination is more complex; speed dropped somewhat when cells were switched from red light to dark conditions (Fig. S5B) and decreased more substantially when switched from green light to dark conditions (Fig. S4A). These differences may be explained by cellular energetics, whereby white light activates photosynthesis more effectively than single wavelengths and therefore cells have more energy. Speed did not drop to zero when switching from green to blue light (Fig. S4B and C), indicating that immediately previous exposure to green light is sufficient to transiently overcome the inhibitory effects of blue light on motility. Thus, our results with different wavelengths show the clear dependence of the motility response on specific photoreceptors, though it is difficult to decouple this response from the photosynthesis-derived energy production that also affects motility. Nevertheless, there is a long deactivation time scale after red/green illumination that is also evident in the reversal of bias after blue light inhibition (Fig. 6C).

Single-cell tracking provides a powerful way to increase our understanding of the molecular pathways by which photoreceptors communicate with and activate type IV pili to mediate a specific phototactic response. For example, we determined that cells exposed to two perpendicular LEDs move in the direction of the vector sum of incidence directions (Fig. 2B and 3). We envision two possibilities for achieving this behavior: (i) the input signals are combined before type IV pilus activation, or (ii) type IV pili pull in two different directions but cellular movement is a product of multiple pulling events, and the force vector that determines motion is along the vector sum. Our previous study (10) suggested that consecutive steps taken by a single cell over 1-s imaging intervals are often due to the retraction of a single pilus, arguing for the first hypothesis above, although if the two pili were synchronized, then our data would be inconclusive. Labeling pili for a direct readout of retraction, or accomplishing an indirect readout via traction force (28), would provide insight into this situation, particularly if combined with the determination of photoreceptor localization via use of fluorescent protein fusions.

The ability of *Synechocystis* cells to sense light directionality is particularly striking, given that their size is on par with the wavelength of visible light. A recent study suggested that directional light sensing is possible in *Synechocystis* cells because they act as spherical microlenses. This allows cells to “see” a light source, because the light is focused on the edge of the cell opposite from the source. This focusing then triggers movement away from the focused spot, resulting in movement toward the light source (9). Our current study suggests that cells sensitively tune their response based on light intensity (Fig. 1 and 4). Responses after exposure to opposing LEDs suggest that cells are able to detect intensity differences, polarizing their motion toward the brighter LED in a position-dependent fashion (Fig. 2 and 3). When exposed to perpendicular LEDs, *Synechocystis* communities started moving in one direction and then turned 90° (Fig. 2B), indicating that the shape of the incident light cone determines the cellular response and that cells can adjust to new light directions rapidly, without residual motion in the original direction of movement.

To the best of our knowledge, the current study is the first to address the integration of light directionalities and wavelengths by *Synechocystis* cells, revealing a broad range of behaviors that lead to regulation and competition among the photoreceptors that collectively contribute to the phototaxis response. The increase in speed when cells are illuminated by two opposing LEDs (Fig. 3B) means that the elimination of motility bias is not due to an inhibition of pilus retraction events. Speed only increased parallel to the light source (Fig. 3B), with larger steps in these directions (Fig. 3D, panel ii), suggesting that exposure to the two LEDs altered the pulling activity along this axis. A previous study with *Neisseria gonorrhoeae* proposed a tug-of-war mechanism in which type IV pili drive persistent motility (29). While the details are likely different for *Synechocystis*, we hypothesize a similar model in which the increased cellular motion back and forth along the light axis creates mechanical tension within the cell envelope that upregulates some part of the process of pilus retraction, such as the frequency of extension/retraction and/or force generation. This model would require that the two opposing LEDs cause pulling in both directions simultaneously, in contrast to the single-LED situation.

In water columns where freshwater *Synechocystis* is normally found, blue and green light are likely to be less absorbed than infrared or red light, and therefore blue and green light may be important light cues. The contrasting enhancement of phototaxis by green light and inhibition by blue light (Fig. 4), coupled to the variable increase in proliferation caused by each (Fig. 4), provides intriguing possibilities for their combined effects. We have provided the first evidence that blue light inhibits phototaxis by virtually inhibiting cell movement (Fig. 4C; Fig. S3). The intensity dependence of the competition between green and blue inputs (Fig. 5) also suggests that none of the photoreceptors has a dominant effect. Nonetheless, reversion of motility bias from blue light occurs over a longer time scale (Fig. 6C and D) than that from other light inputs we measured, especially reversion from the dark (Fig. 6B). Blue light inactivates TaxD1, whose presence promotes positive phototaxis (5, 8); however, blue light illumination does not have the same effect as deletion of TaxD1, since we did not observe any negative phototaxis (Fig. 4C). This difference likely occurs because blue light also turns on blue light-responsive photoreceptors, such as Cph2, PixD1, and UirS (7, 16, 19, 26), suggesting that analysis of the motility of mutants (potentially multiple knockouts) under varied light conditions may enable further deconstruction of the blue light response in *Synechocystis*.

Here, an important component of the combination of green and blue light was the degree of cell proliferation, which drove a switch from complete inhibition of motility at high intensity to an increase in phototaxis at intermediate intensity (Fig. 5). Given that blue light appears to stimulate more proliferation and inhibit motility (Fig. 4C), it is intriguing to consider the possibility of negative feedback between proliferation and motility in *Synechocystis*, as has been observed in the swarming bacteria *Pseudomonas aeruginosa* (30) and *Myxococcus xanthus* (31). While there may be similar feedback in *Synechocystis*, such a stark switch between motility and proliferation cannot exist, as evidenced by the coupled increase in phototaxis and proliferation under green light illumination (Fig. 4); perhaps motility is aided by proliferation due to the requirement for high EPS levels. Moreover, since speed is partially maintained when switching from green to blue light (Fig. S4B and C), the time scales of exposure to a particular wavelength must also matter and not just whether photoreceptors are activated; this effect may be due to the effects of green and blue light on proliferation. Most models of *Synechocystis* motility have neglected proliferation, as it is difficult to measure since it must be measured as a function of wavelength, on a surface, and as it varies in time and space. Microfluidics may be necessary to rapidly probe this parameter space, and quantitative analyses of community phenotypes could be used to infer the pattern of cell division and growth.

Phototaxis represents both a signaling and a motility response, requiring consideration at the single-cell and community scales. Given that cyanobacteria routinely experience complex environments, it is critical to establish experimental systems, such as the one employed here, that enable tuning of the light input in terms of wavelength, flux, and directionality. The current study highlights the capacity of *Synechocystis* cells to integrate multiple inputs, rather than simply choosing to respond to a light signal that dominates other signals. This integration of signals likely occurs within and outside the cell through the interaction of signaling pathways with their photoreceptors and through competition among pili across the cell surface. Motility also depends on other factors, such as EPS concentration, which is determined by the local cell density (11); hence, motility also depends on the promotion of cellular proliferation by the light environment. Therefore, phototaxis on the community scale is a collective behavior that relies on a combination of signal transduction of a light input, surface sensing through EPS-mediated mobility enhancement, mechanical feedback on pilus activity, and the local and global effects of cell density. These connections can be generalized to other multicellular behaviors in which dynamic interactions among multiple factors over various time scales, length scales, and/or modes of action (chemical/mechanical/energetic) are critical for comprehending the emergent properties of the community.

## MATERIALS AND METHODS

### Strains and growth conditions.

*Synechocystis* sp. PCC6803 cells from a strain collection at the Carnegie Institution at Stanford were grown from an original single colony of phototaxis-positive cells in BG-11 medium (32) at 30°C with continuous shaking at 100 rpm under overhead warm white fluorescent light (Super Saver Warm white; F40WW/SS; 34 W). The incident fluence rate was 10 μE m^−2^ s^−1^. All imaging experiments were performed using exponentially growing cells with an optical density at 730 nm (OD_730_) of 0.8 (33,000 cells/μl) as measured with an Ultrospec 3100 Pro spectrophotometer (Amersham Biosciences, Inc.).

### Motility assays.

Motility assays were carried out on 0.4% (wt/vol) agarose in BG-11 medium in 50-mm plastic petri dishes (BD Falcon) or 100-mm square dishes (Simport, Quebec, Canada) at 30°C. One microliter of cells from a culture with an OD_730_ of 0.8 was placed at a particular position on a plate, and then the plate was inverted to minimize evaporation of the agarose. One or two LEDs (Roithner LaserTechnik GmbH) of the appropriate wavelength(s) (red: LED660N-03, 5 mm, 15 mW, 24° spread; green: LED535-01, 5 mm, 4 mW, 8° spread; blue: LED435-03, 5 mm, 20 mW, 12° spread) were used to illuminate the plate. To induce phototaxis, the LED(s) was placed 50 mm from the center of the center-most droplet (which had a diameter of 2.5 mm) at the level of the agarose (Fig. 1 to 3), or at 10 increasing distances (starting at 11.4 mm, with increments of 5.7 mm) away from the centers of the inoculations at the level of the agarose (Fig. 4 and 5).

### Measurement of incident light intensities.

Incident light intensities were measured along the direction tangent to the light vector of the relevant LED(s) at the positions of inoculations by using a Black-Comet UV-Vis spectrometer (StellarNet Inc.).

### Time-lapse imaging.

Entire droplets of cells were imaged using a Canon 60D DSLR camera (Canon United States, Inc.) attached to an MZ12 stereoscope (Leica Microsystems, Inc.). In light manipulation experiments, cells were placed under directional light for at least 24 h until finger-like projections had formed from the site of inoculation. In the light on/off experiment, the power supply to the LED was turned off or on, depending on the desired light condition. Time-lapse imaging at single-cell resolution was conducted at 20× magnification and 1-frame/s intervals by using a Coolsnap-Pro monochrome camera (Photometrics) attached to a TE-300 inverted microscope (Nikon Instruments Inc.). The temperature was maintained at 30°C in all time-lapse imaging experiments by use of a custom environmental chamber (HaisonTech).

### Cell tracking and analysis.

Cell tracking was performed using custom MatLab software (MathWorks) (10) to quantify the positions and velocities of individual cells over time. In each frame, individual cells were segmented using thresholding and a watershed transform, and the locations of their centers of mass were recorded. To avoid artifacts due to inconsistent segmentation of cell doublets, centroids that were closer than 0.93 μm were removed from subsequent analyses and the tracks of the associated cells were terminated. The track of each cell was identified using the nearest-neighbor distance across frames. The step size taken over each 1-s interval between frames was defined as the Euclidean distance moved by the cell over that time. To avoid incorrect assignment of tracks at high cell density, step sizes of >1.86 μm were ignored and those tracks were terminated. The step angle was calculated relative to the light axis by taking the inverse tangent of the ratio between the displacements along the perpendicular and parallel axes. Steps taken directly toward or away from the light were defined as 0° and 180°, respectively. The speed, velocity, and bias values of each cell were calculated over 100-s intervals, unless otherwise indicated. These parameters were measured separately along the two axes (parallel and perpendicular to the light) and included the times during those intervals when cells appeared stationary. Speed and velocity were calculated by dividing the total path length of displacement and the displacement along the relevant axis, respectively, by the length of the time interval of measurement. Movement bias was calculated by dividing the resultant displacement by the total path length.

### Data availability.

Data in our article’s figures include time-lapse microscopy images of single cells and images of whole droplets of cells. All images are available upon request from the corresponding author.

## References

[1] Mullineaux CW 2001 How do cyanobacteria sense and respond to light? Mol Microbiol 41:965–971. doi:10.1046/j.1365-2958.2001.02569.x. 11555279

[2] Schweinitzer T, Josenhans C 2010 Bacterial energy taxis: a global strategy? Arch Microbiol 192:507–520. doi:10.1007/s00203-010-0575-7. 20411245 PMC2886117

[3] Van Haastert PJ, Devreotes PN 2004 Chemotaxis: signalling the way forward. Nat Rev Mol Cell Biol 5:626–634. doi:10.1038/nrm1435. 15366706

[4] Bhaya D 2004 Light matters: phototaxis and signal transduction in unicellular cyanobacteria. Mol Microbiol 53:745–754. doi:10.1111/j.1365-2958.2004.04160.x. 15255889

[5] Yoshihara S, Ikeuchi M 2004 Phototactic motility in the unicellular cyanobacterium *Synechocystis* sp. PCC 6803. Photochem Photobiol Sci 3:512–518. doi:10.1039/b402320j. 15170479

[6] Choi JS, Chung YH, Moon YJ, Kim C, Watanabe M, Song PS, Joe CO, Bogorad L, Park YM 1999 Photomovement of the gliding cyanobacterium *Synechocystis* sp. PCC 6803. Photochem Photobiol 70:95–102. doi:10.1111/j.1751-1097.1999.tb01954.x. 10420848

[7] Moon YJ, Kim SI, Chung YH 2012 Sensing and responding to UV-A in cyanobacteria. Int J Mol Sci 13:16303–16332. doi:10.3390/ijms131216303. 23208372 PMC3546692

[8] Ng WO, Grossman AR, Bhaya D 2003 Multiple light inputs control phototaxis in *Synechocystis* sp. strain PCC6803. J Bacteriol 185:1599–1607. doi:10.1128/JB.185.5.1599-1607.2003. 12591877 PMC148062

[9] Schuergers N, Lenn T, Kampmann R, Meissner MV, Esteves T, Temerinac-Ott M, Korvink JG, Lowe AR, Mullineaux CW, Wilde A 2016 Cyanobacteria use micro-optics to sense light direction. eLife 5:e12620. doi:10.7554/eLife.12620. 26858197 PMC4758948

[10] Chau RM, Ursell T, Wang S, Huang KC, Bhaya D 2015 Maintenance of motility bias during cyanobacterial phototaxis. Biophys J 108:1623–1632. doi:10.1016/j.bpj.2015.01.042. 25863054 PMC4390813

[11] Burriesci M, Bhaya D 2008 Tracking phototactic responses and modeling motility of *Synechocystis* sp. strain PCC6803. J Photochem Photobiol B 91:77–86. doi:10.1016/j.jphotobiol.2008.01.012. 18343151

[12] Ursell T, Chau RM, Wisen S, Bhaya D, Huang KC 2013 Motility enhancement through surface modification is sufficient for cyanobacterial community organization during phototaxis. PLoS Comput Biol 9:e1003205. doi:10.1371/journal.pcbi.1003205. 24039562 PMC3763999

[13] Skerker JM, Berg HC 2001 Direct observation of extension and retraction of type IV pili. Proc Natl Acad Sci U S A 98:6901–6904. doi:10.1073/pnas.121171698. 11381130 PMC34450

[14] Chiang P, Habash M, Burrows LL 2005 Disparate subcellular localization patterns of *Pseudomonas aeruginosa* type IV pilus ATPases involved in twitching motility. J Bacteriol 187:829–839. doi:10.1128/JB.187.3.829-839.2005. 15659660 PMC545728

[15] Burrows LL 2012 *Pseudomonas aeruginosa* twitching motility: type IV pili in action. Annu Rev Microbiol 66:493–520. doi:10.1146/annurev-micro-092611-150055. 22746331

[16] Fiedler B, Börner T, Wilde A 2005 Phototaxis in the cyanobacterium *Synechocystis* sp. PCC 6803: role of different photoreceptors. Photochem Photobiol 81:1481–1488. doi:10.1562/2005-06-28-RA-592. 16354116

[17] Bhaya D, Takahashi A, Shahi P, Grossman AR 2001 Novel motility mutants of *Synechocystis* strain PCC 6803 generated by in vitro transposon mutagenesis. J Bacteriol 183:6140–6143. doi:10.1128/JB.183.20.6140-6143.2001. 11567015 PMC99694

[18] Yoshihara S, Suzuki F, Fujita H, Geng XX, Ikeuchi M 2000 Novel putative photoreceptor and regulatory genes required for the positive phototactic movement of the unicellular motile cyanobacterium *Synechocystis* sp. PCC 6803. Plant Cell Physiol 41:1299–1304. doi:10.1093/pcp/pce010. 11134414

[19] Song JY, Cho HS, Cho JI, Jeon JS, Lagarias JC, Park YI 2011 Near-UV cyanobacteriochrome signaling system elicits negative phototaxis in the cyanobacterium *Synechocystis* sp. PCC 6803. Proc Natl Acad Sci U S A 108:10780–10785. doi:10.1073/pnas.1104242108. 21670284 PMC3127908

[20] Bhaya D, Nakasugi K, Fazeli F, Burriesci MS 2006 Phototaxis and impaired motility in adenylyl cyclase and cyclase receptor protein mutants of *Synechocystis* sp. strain PCC 6803. J Bacteriol 188:7306–7310. doi:10.1128/JB.00573-06. 17015670 PMC1636242

[21] Bhaya D, Watanabe N, Ogawa T, Grossman AR 1999 The role of an alternative sigma factor in motility and pilus formation in the cyanobacterium *Synechocystis* sp. strain PCC 6803. Proc Natl Acad Sci U S A 96:3188–3193. doi:10.1073/pnas.96.6.3188. 10077659 PMC15917

[22] Bhaya D, Bianco NR, Bryant D, Grossman A 2000 Type IV pilus biogenesis and motility in the cyanobacterium *Synechocystis* sp. PCC 6803. Mol Microbiol 37:941–951. doi:10.1046/j.1365-2958.2000.02068.x. 10972813

[23] Mattick JS 2002 Type IV pili and twitching motility. Annu Rev Microbiol 56:289–314. doi:10.1146/annurev.micro.56.012302.160938. 12142488

[24] Yoshihara S, Katayama M, Geng X, Ikeuchi M 2004 Cyanobacterial phytochrome-like PixJ1 holoprotein shows novel reversible photoconversion between blue- and green-absorbing forms. Plant Cell Physiol 45:1729–1737. doi:10.1093/pcp/pch214. 15653792

[25] Okajima K, Yoshihara S, Fukushima Y, Geng X, Katayama M, Higashi S, Watanabe M, Sato S, Tabata S, Shibata Y, Itoh S, Ikeuchi M 2005 Biochemical and functional characterization of BLUF-type flavin-binding proteins of two species of cyanobacteria. J Biochem 137:741–750. doi:10.1093/jb/mvi089. 16002996

[26] Wilde A, Fiedler B, Börner T 2002 The cyanobacterial phytochrome Cph2 inhibits phototaxis towards blue light. Mol Microbiol 44:981–988. doi:10.1046/j.1365-2958.2002.02923.x. 12010493

[27] Tanaka K, Nakasone Y, Okajima K, Ikeuchi M, Tokutomi S, Terazima M 2012 Time-resolved tracking of interprotein signal transduction: *Synechocystis* PixD-PixE complex as a sensor of light intensity. J Am Chem Soc 134:8336–8339. doi:10.1021/ja301540r. 22563901

[28] Munevar S, Wang Y, Dembo M 2001 Traction force microscopy of migrating normal and H-ras transformed 3T3 fibroblasts. Biophys J 80:1744–1757. doi:10.1016/S0006-3495(01)76145-0. 11259288 PMC1301364

[29] Marathe R, Meel C, Schmidt NC, Dewenter L, Kurre R, Greune L, Schmidt MA, Müller MJ, Lipowsky R, Maier B, Klumpp S 2014 Bacterial twitching motility is coordinated by a two-dimensional tug-of-war with directional memory. Nat Commun 5:3759. doi:10.1038/ncomms4759. 24806757

[30] Bains M, Fernández L, Hancock RE 2012 Phosphate starvation promotes swarming motility and cytotoxicity of *Pseudomonas aeruginosa*. Appl Environ Microbiol 78:6762–6768. doi:10.1128/AEM.01015-12. 22773629 PMC3426718

[31] Claessen D, Rozen DE, Kuipers OP, Søgaard-Andersen L, van Wezel GP 2014 Bacterial solutions to multicellularity: a tale of biofilms, filaments and fruiting bodies. Nat Rev Microbiol 12:115–124. doi:10.1038/nrmicro3178. 24384602

[32] Stanier RY, Kunisawa R, Mandel M, Cohen-Bazire G 1971 Purification and properties of unicellular blue-green algae (order Chroococcales). Bacteriol Rev 35:171–205. 4998365 10.1128/br.35.2.171-205.1971 PMC378380

