# Supplementary figures and images for: Emergent Phototactic Responses of Cyanobacteria under Complex Light Regimes
Source: mBio. 2017 Mar 7;8(2):e02330-16. doi: 10.1128/mBio.02330-16 (PMC5340875; doi:10.1128/mBio.02330-16)

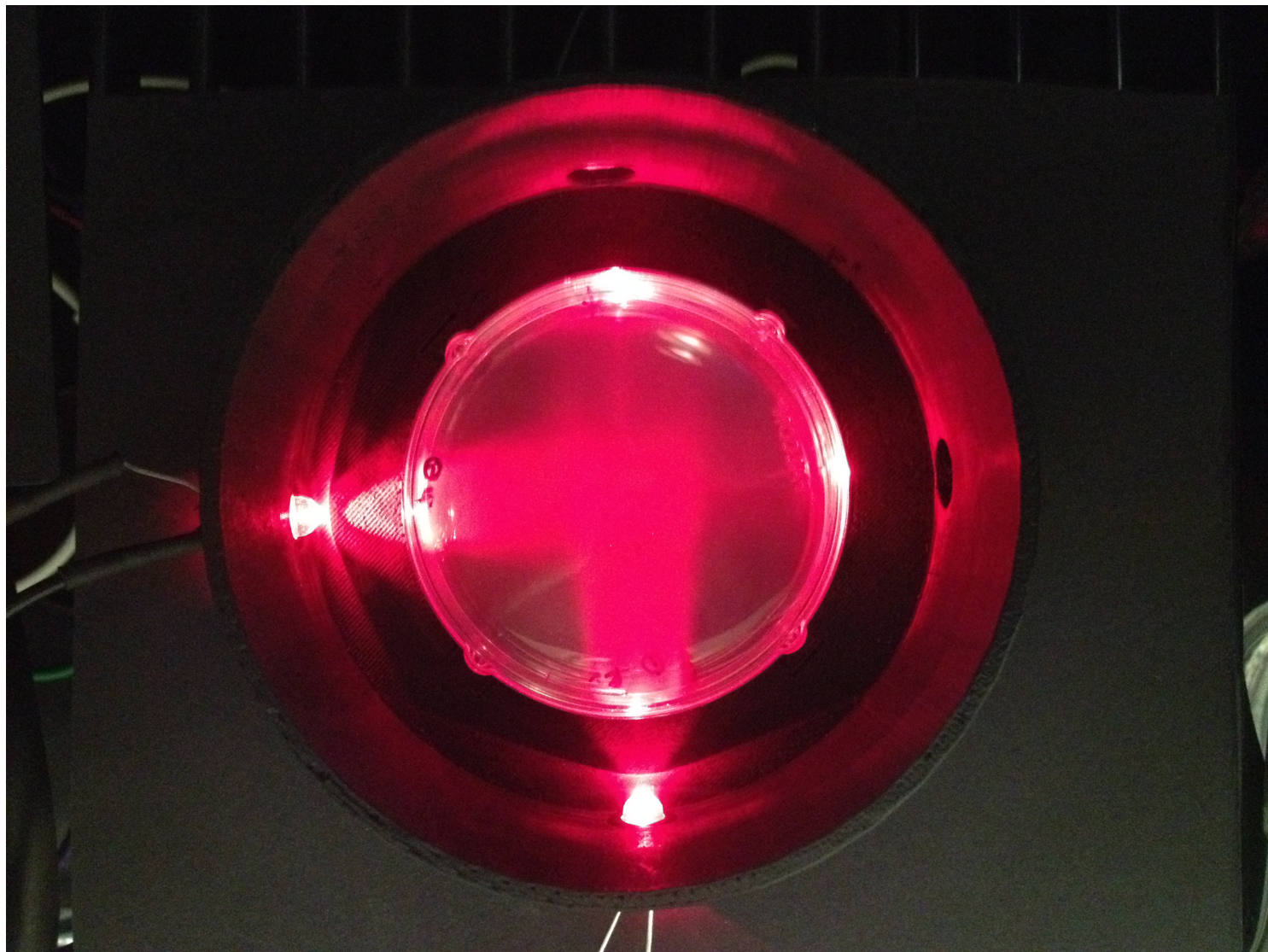

Supplement: FIG S1 [file mbo001173188sf1.pdf]

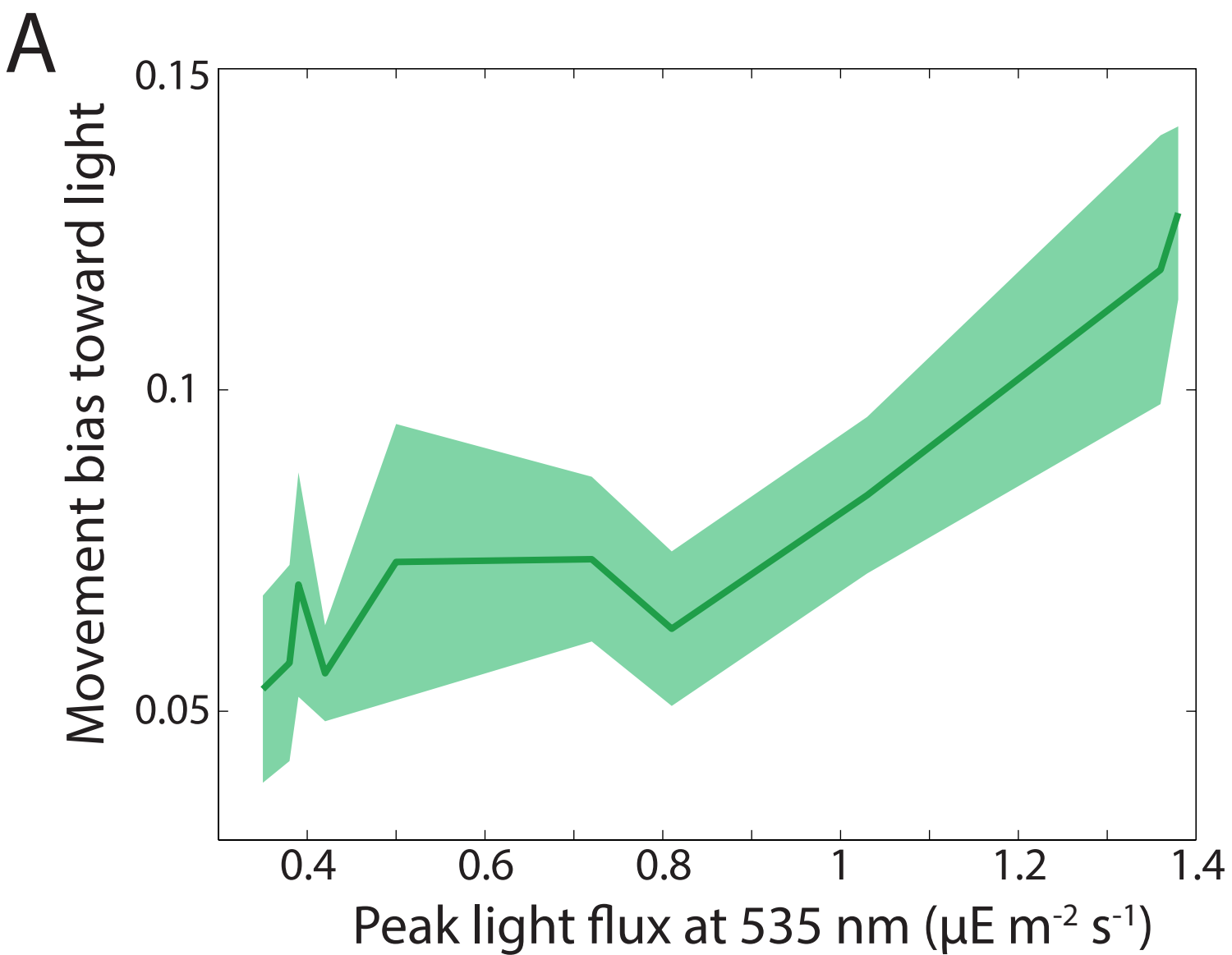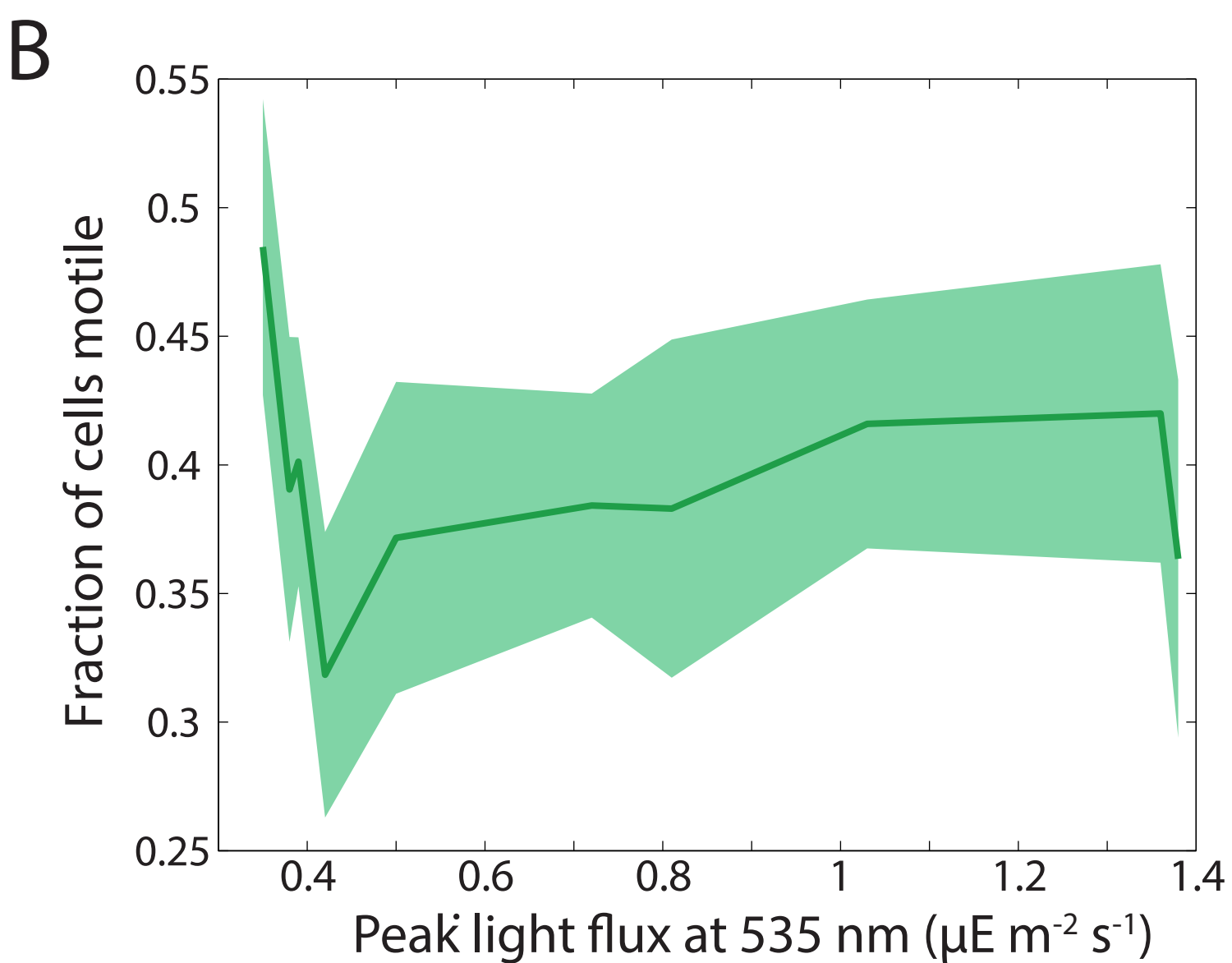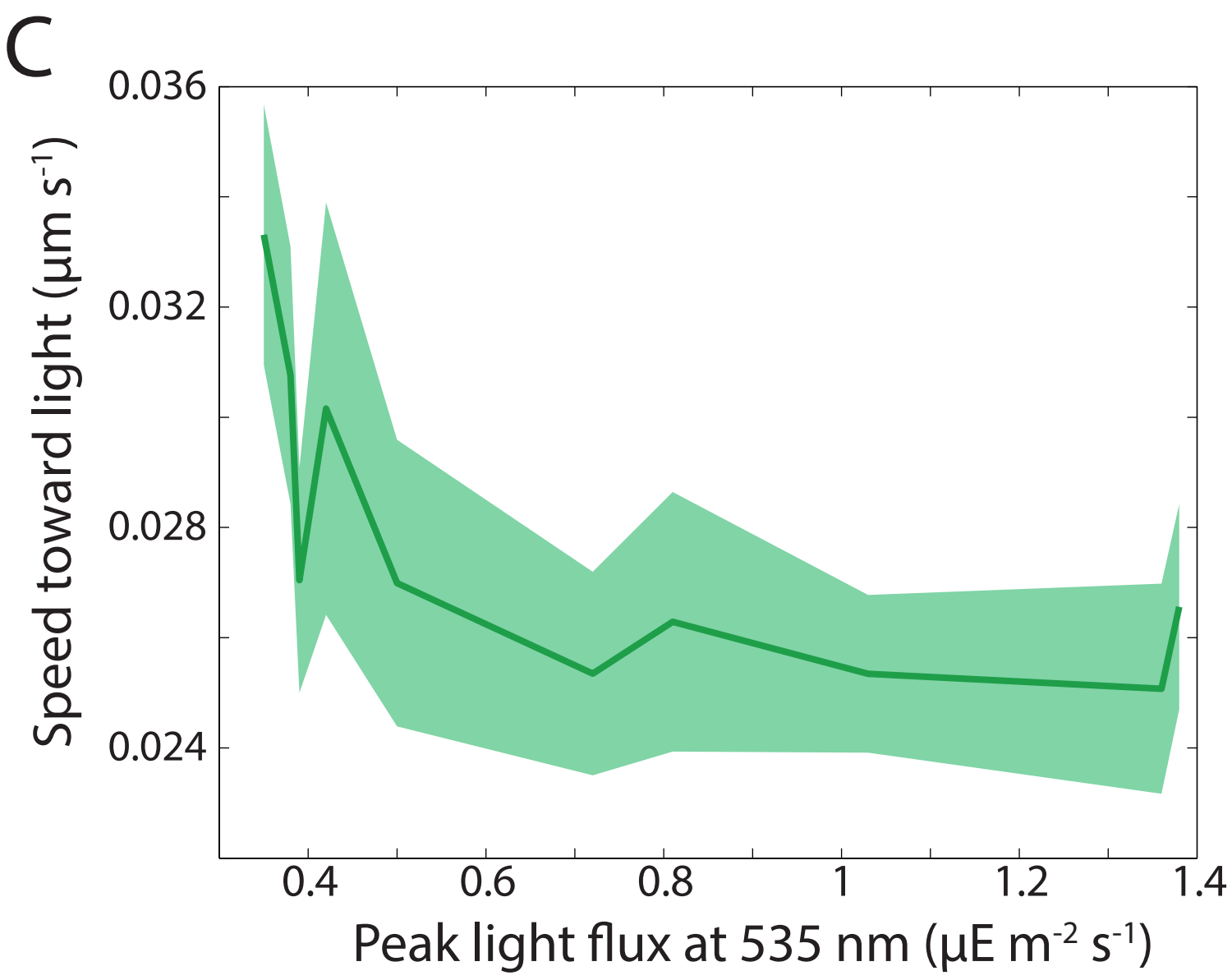

Supplement: FIG S2 [file mbo001173188sf2.pdf]

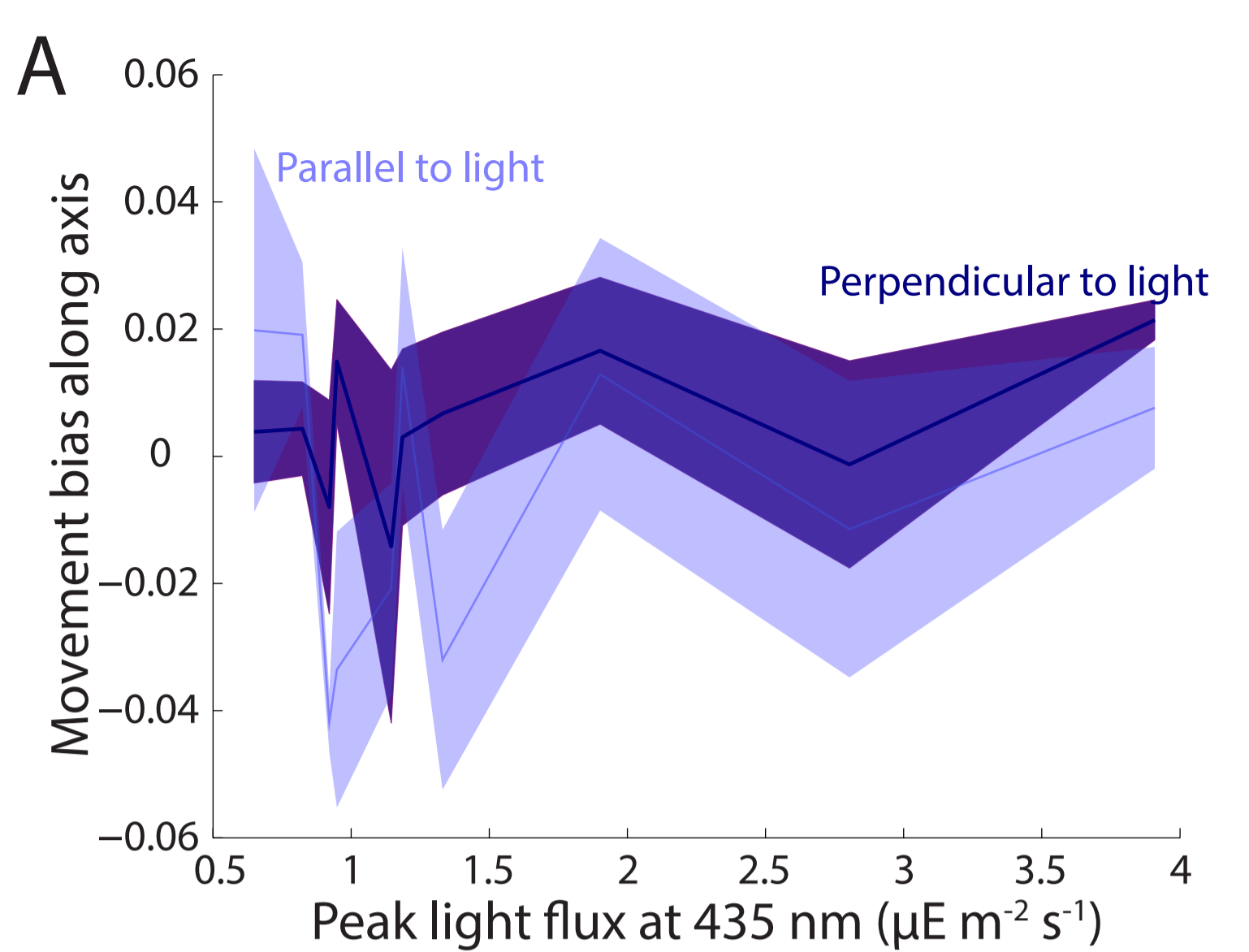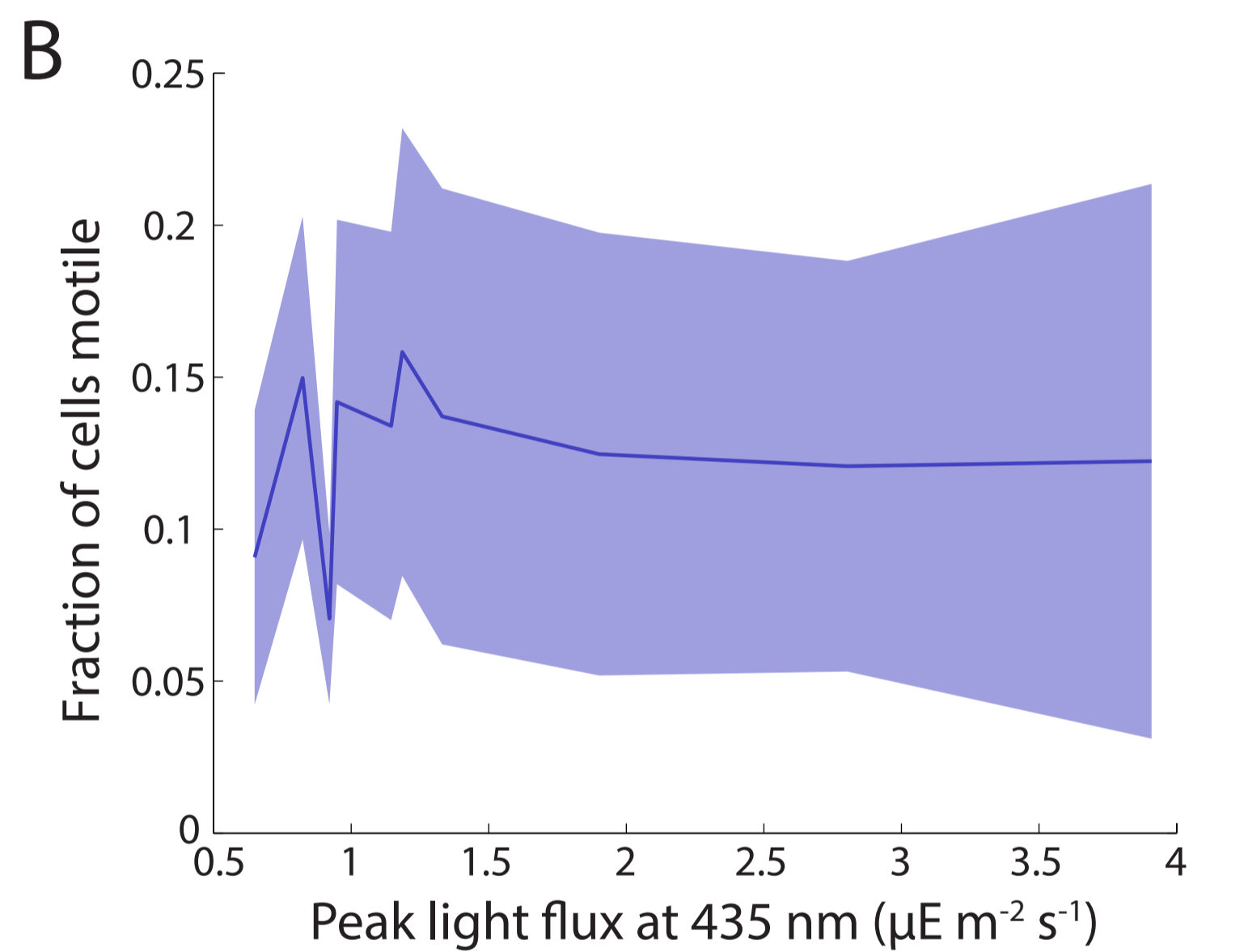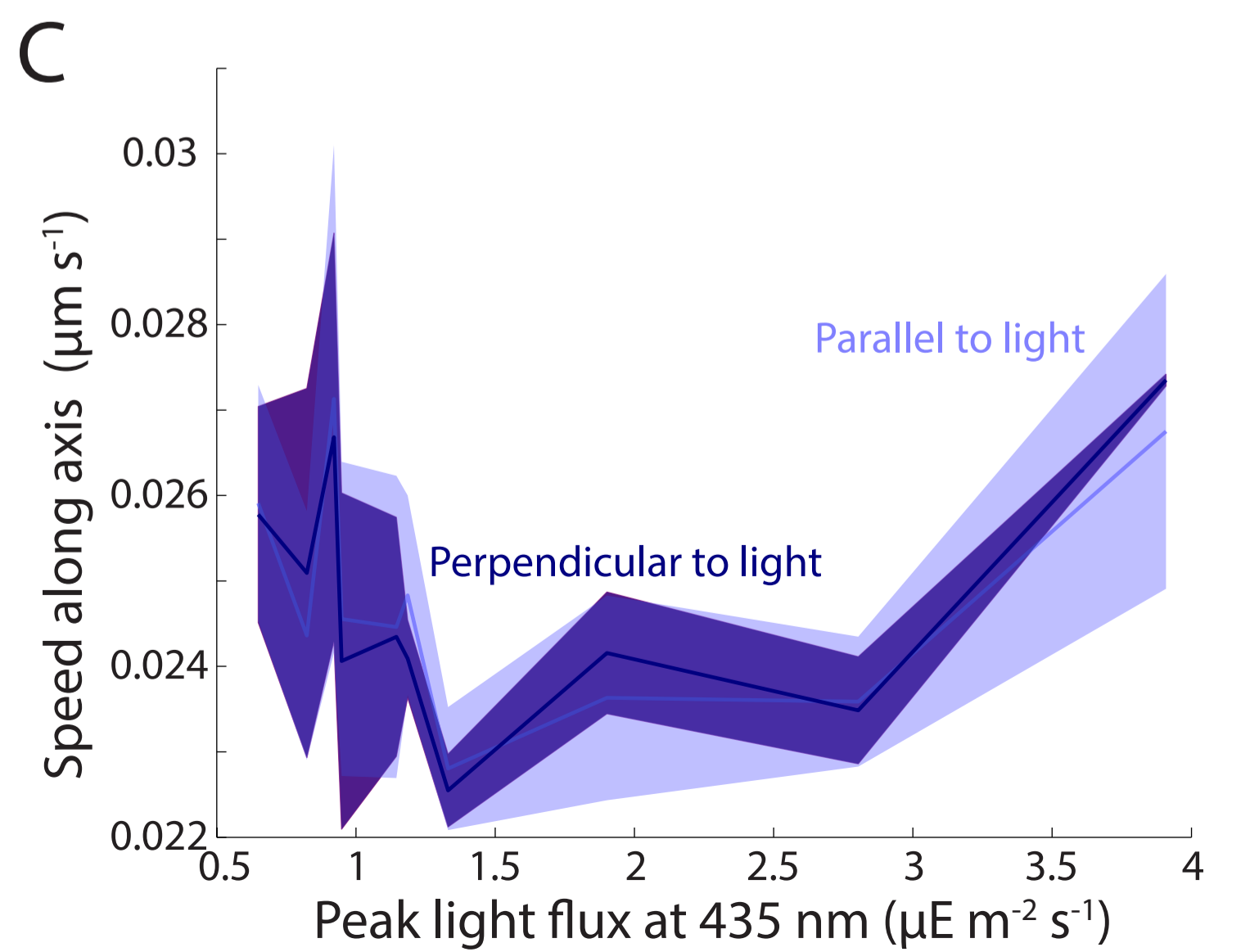

Supplement: FIG S3 [file mbo001173188sf3.pdf]

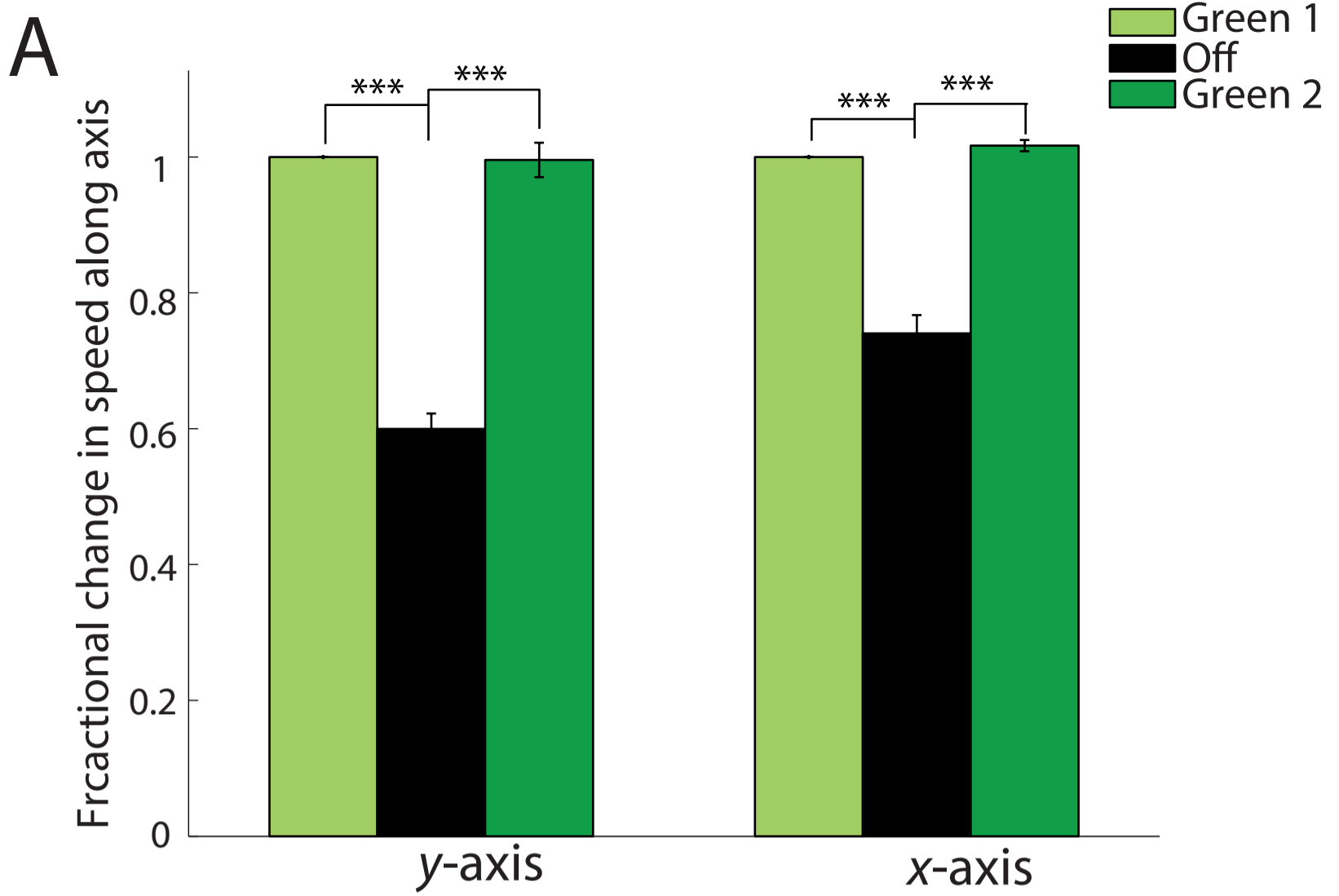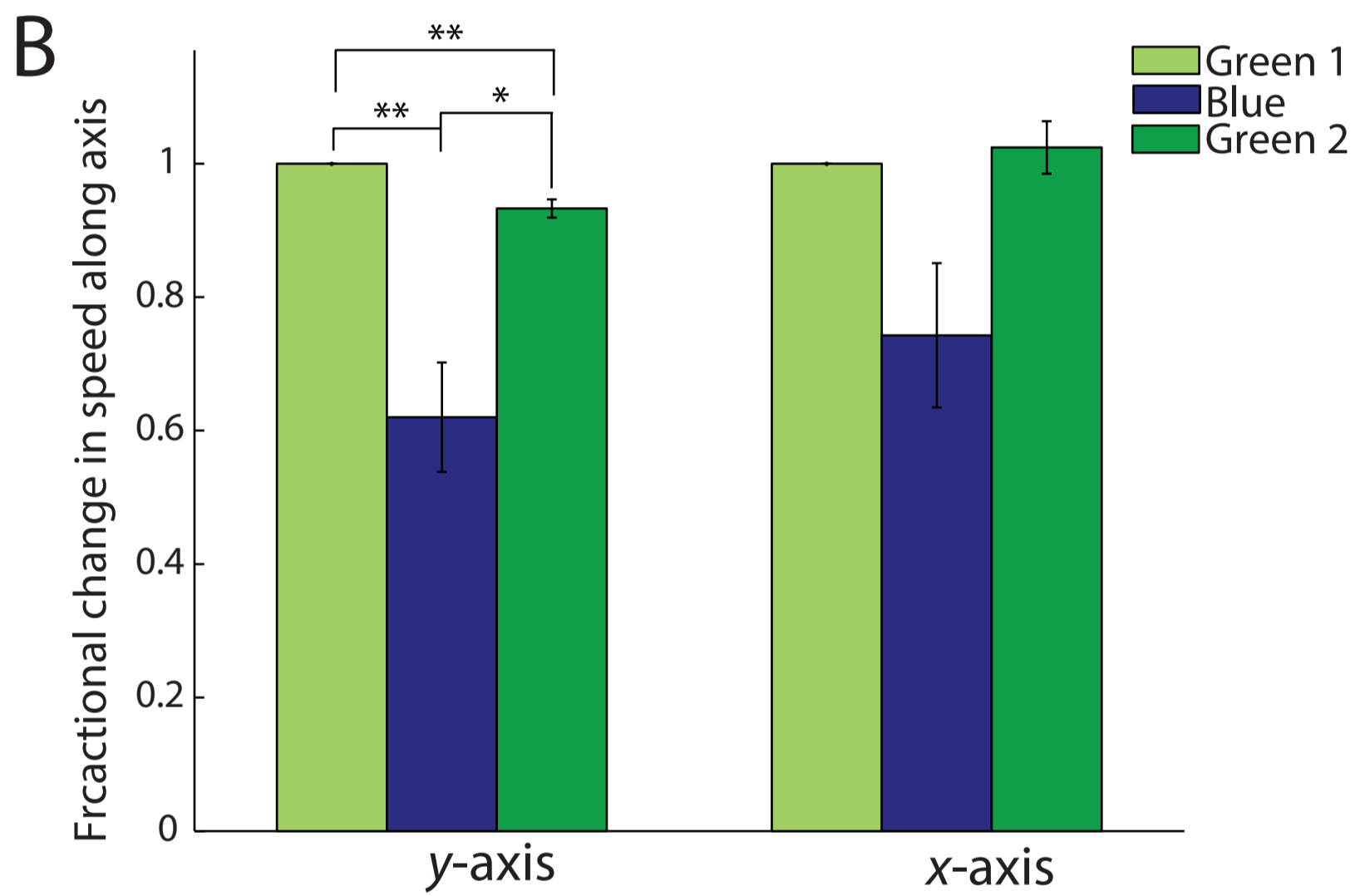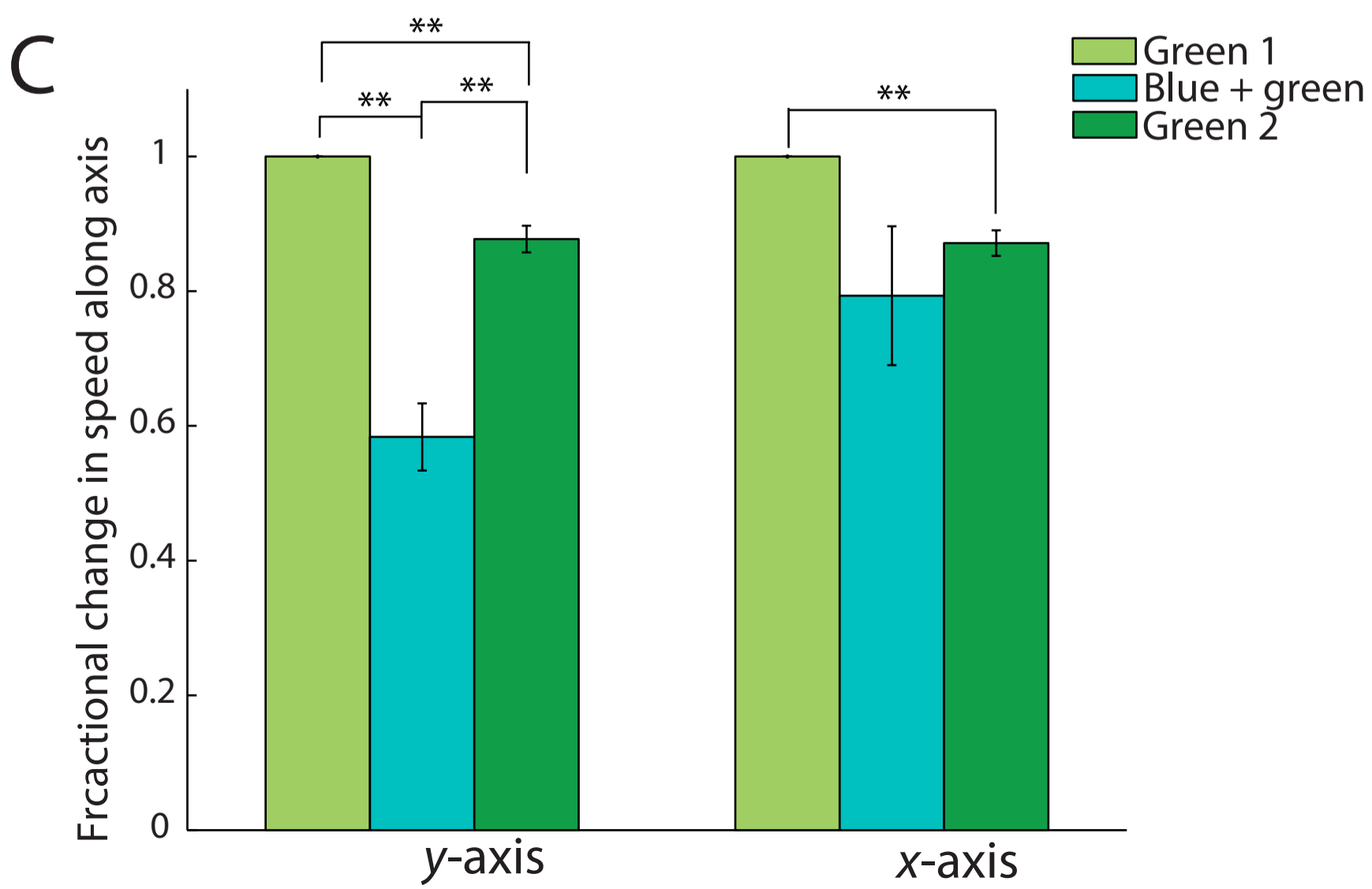

Supplement: FIG S4 [file mbo001173188sf4.pdf]

**A**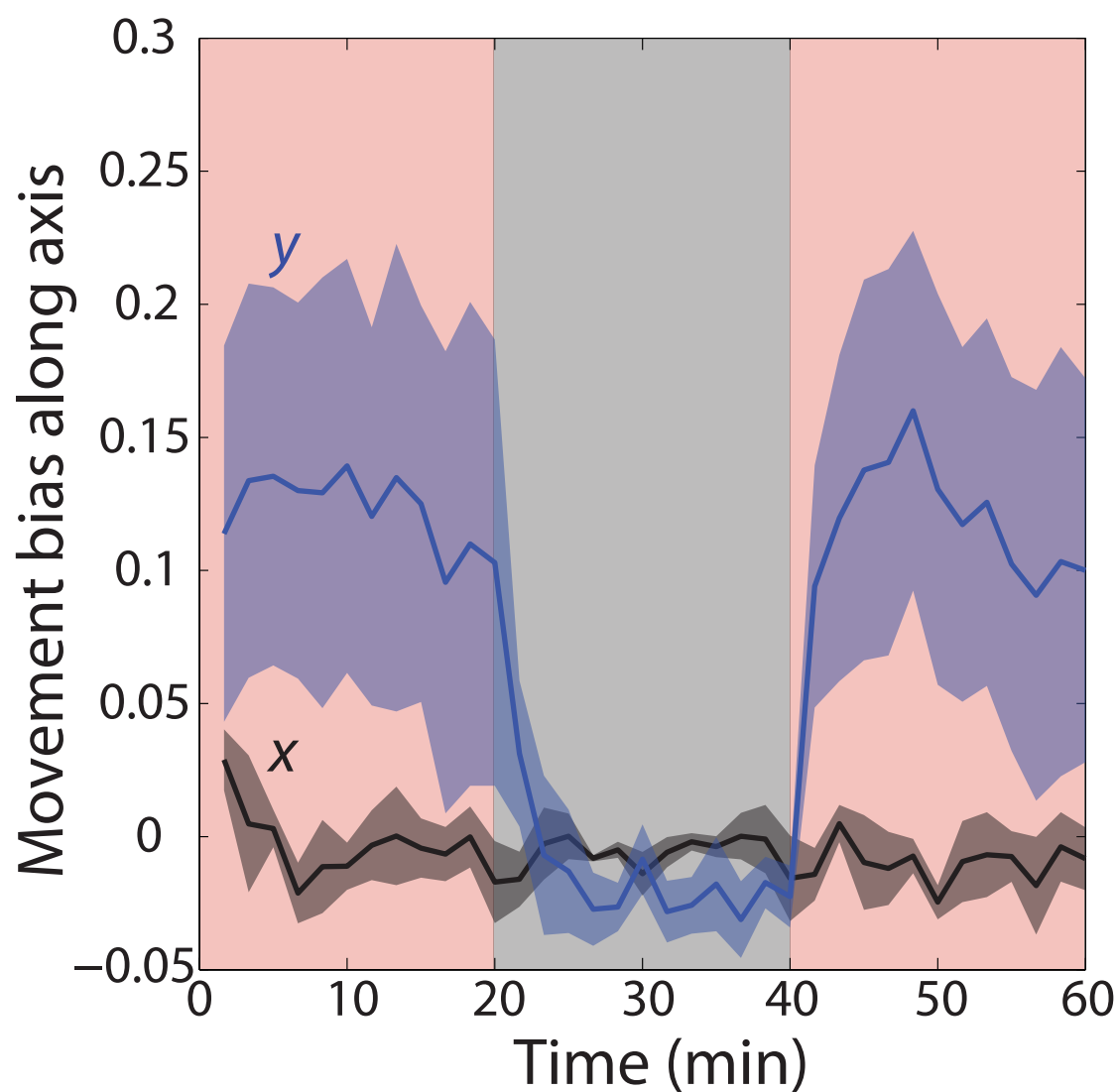**B**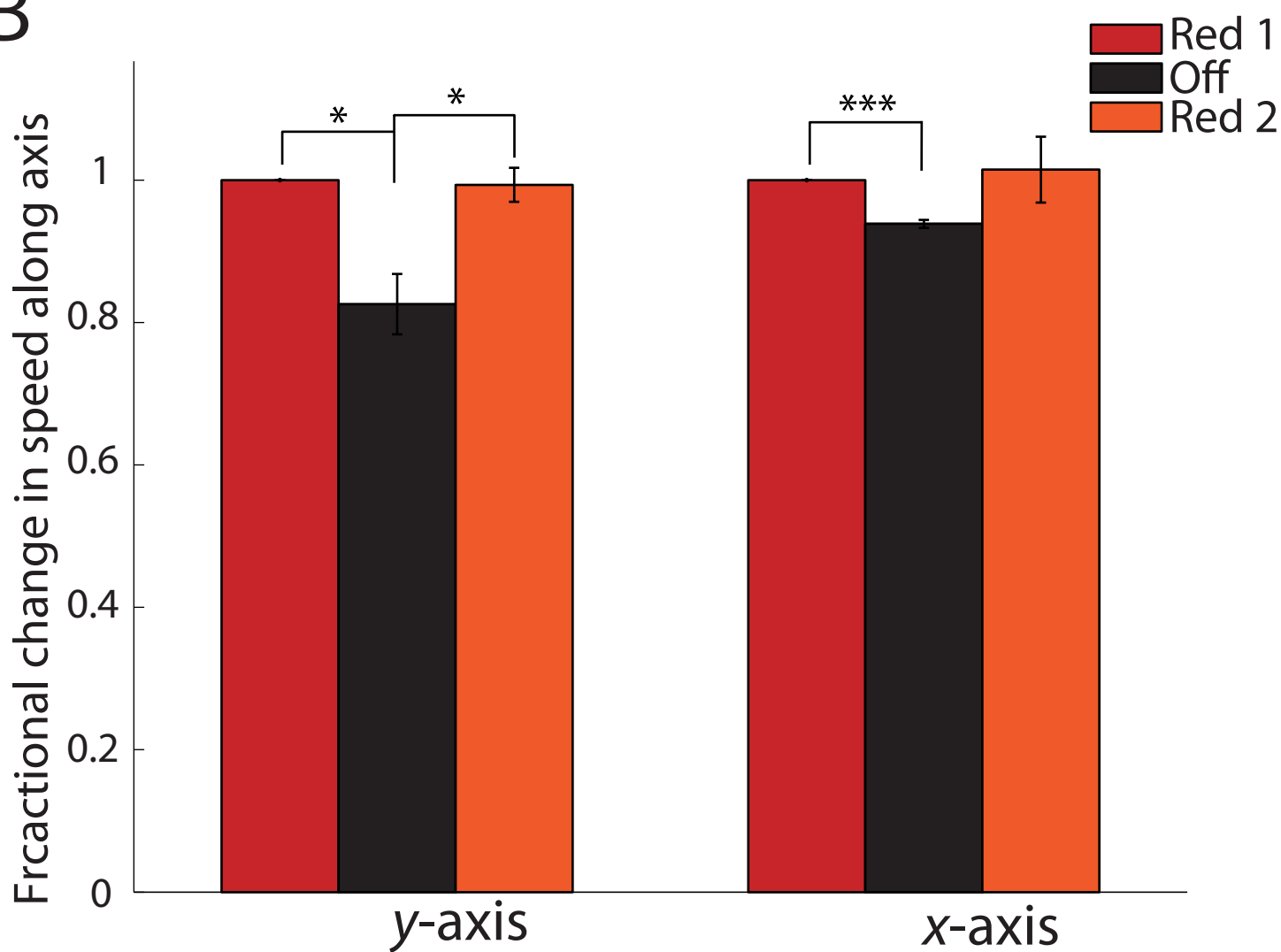

Supplement: FIG S5 [file mbo001173188sf5.pdf]
